# Improving Brain Tumor Detection by Cortical Surface and Vessels Segmentation Through RGB-to-HSI Transfer Learning

**DOI:** 10.3390/cancers18050857

**Published:** 2026-03-06

**Authors:** Guillermo Vazquez, Alberto Martín-Pérez, Angel Perez-Nuñez, Alfonso Lagares, Eduardo Juarez, Cesar Sanz

**Affiliations:** 1 Research Center on Software Technologies and Multimedia Systems, Universidad Politécnica de Madrid (UPM), 28031 Madrid, Spain; a.martinp@upm.es (A.M.-P.);; 2 Neurosurgery Department, Hospital Universitario 12 de Octubre, 28041 Madrid, Spain; 3 Medicine Faculty, Universidad Complutense de Madrid (UCM), 28040 Madrid, Spain; 4 Instituto de Investigación Sanitaria Hospital 12 de Octubre (Imas12), 28041 Madrid, Spain

**Keywords:** hyperspectral, segmentation, transfer learning, vessel segmentation, cortical segmentation

## Abstract

**Background**: Accurate in vivo brain tumor detection using hyperspectral imaging (HSI), a non-invasive technique that captures spectral information beyond the visible range, is challenging due to the complexity of biological tissues and the difficulty in distinguishing malignant from healthy areas. Conventional neural-network-based methods often misclassify tumor tissue as blood vessels, largely due to high vascularization and the scarcity of annotated data. **Method**: To address this issue, this work proposes an underexplored approach that decomposes the problem into two tasks: (1) segmentation of the brain cortical surface and its blood vessels, and (2) segmentation of biological tissues within the segmented craniotomy site. The cortical segmentation task is addressed independently of the segmentation model used in the second stage. To achieve this, a set of pseudo-labels is generated from RGB and HSI captures acquired during in vivo brain surgeries. These pseudo-labels support a multimodal training strategy that leverages both imaging domains, yielding a model capable of segmenting the craniotomy site and the blood vessels contained in it. The model is further refined on HSI using weakly supervised fine-tuning with sparse ground truth annotations. **Results**: The final segmentation map combines cortical and tissue segmentation outputs, considering only cortex pixels not overlapped by vessels as potential tumor regions. This simplifies the HSI tissue segmentation task, reframing it as a binary segmentation of healthy vs. other tissues, while still enabling a comprehensive multiclass output. **Conclusions**: The proposed method achieves up to a 15.48% increase in F1 score for the tumor class, while segmenting the brain cortex with a mean Dice similarity coefficient (DSC) of 92.08% and accurately detecting 95.42% of labeled blood vessel samples in the HSI dataset.

## Simple Summary

Precise delimitation of brain tumors during surgical intervention remains challenging. Hyperspectral imaging, which captures information beyond the visible spectrum, can be a valuable tool for identifying biological tissues when combined with deep learning algorithms. However, artificial-intelligence-based methods often struggle to distinguish malignant areas from highly vascularized structures, leading to potential misclassification. To address this limitation, we propose a two-stage segmentation strategy where a model first identifies the exposed brain surface and blood vessels. Then, a secondary model classifies pixels from the remaining tissue. To train these models, a set of pseudo-labels is generated with minimal manual intervention using both RGB and hyperspectral images acquired during surgical procedures. By segmenting the brain cortex and its vessels, the proposed approach simplifies the multitissue classification into a binary classification of healthy versus non-healthy tissue. This strategy improves tumor segmentation accuracy, exploring the potential use of hyperspectral imaging for real-time intraoperative brain tumor guidance.

## 1. Introduction

Computer-assisted diagnosis (CAD) is a discipline that has gained importance in recent decades in the medical field, particularly with the development of systems supported by machine learning (ML) and deep learning (DL) techniques [1]. In addition, emerging medical imaging sources, such as HSI, have been incorporated into this algorithmic expansion to increase the analysis capability required in diagnosis processes [2,3]. When this diagnosis focuses on the detection of areas of the brain surface affected by a tumor, HSI offers a non-invasive solution to the differentiation of the tissues present in the brain cortex. In this way, the spatial and spectral information captured by the HS camera provides characteristics of the scene that are beyond the visual spectrum.

Following the current trend of using DL-based methods, established for their proven effectiveness [4], many neural network (NN) solutions have been developed to segment and process HS information with the intention of performing organ identification [5] and tumor segmentation. In relation to the latter objective, significant efforts have been dedicated to refine and improve NN-based models to achieve precise tumor detection and delineation in the form of a clear segmentation map that can be useful to neurosurgeons [6]. However, achieving accurate segmentation of tumor-affected regions remains a challenge. The work presented by Urbanos et al. [7] exemplifies how the difficulty mainly arises from the propensity of the algorithms to misidentify tumor tissue as blood vessels, a problem likely attributable to the high vascularization typical of tumors. In addition to this problem, it should be noted that, as is common in the medical field, it is difficult to have a fully annotated dataset available. The time-consuming nature of the labeling process often results in annotations that are sparse or incomplete, as this is the most feasible way to obtain ground truth data.

In light of this matter, the present work develops a relatively unexplored approach focused on improving tumor detection by reducing the complexity of the problem by splitting it into two different tasks: (1) the segmentation of the cortical surface and the blood vessels present on it, and (2) the segmentation of biological tissues within the area identified as the brain parenchyma. These two tasks are addressed in accordance with their intrinsic characteristics: elements that are easily recognizable to the human eye, such as the boundaries of the brain surface and vascular structures, are intended to be detected focusing on their morphology. In contrast, the distinction between healthy and tumor tissue is aimed at being achieved using the differences in their spectral signatures. The research conducted in this paper proposes a solution for task number (1).

**Figure 1:**
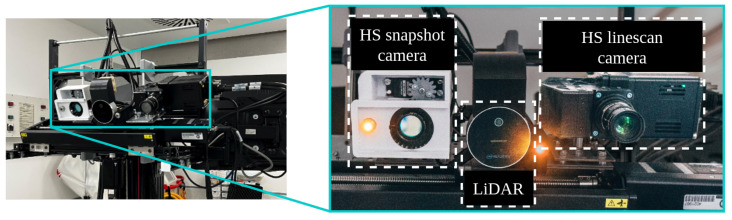
Front view of the *SLIMBRAIN prototype 3* acquisition system, displaying, from left to right, the HS snapshot camera, the Intel L515 LiDAR, and the HS linescan camera, all three mounted on the Zaber linear stage. Lower image is reproduced from [8] under license CC BY-NC-ND 4.0.

The proposed method utilizes HS and RGB imaging sources obtained from in vivo brain tumor surgeries at the University Hospital 12 de Octubre in Madrid, Spain. The image acquisition system used, illustrated in Figure 1 and described later in Section 3.1, integrates a snapshot HS camera and a LiDAR device with a time-of-flight (ToF) depth sensor and an RGB camera. As the HS snapshot camera is capable of streaming HS video, it is possible to perform a real-time segmentation of the biological tissues present in the scene. Combining the depth information with the tissue segmentation performed on the HS stream, the system produces a three-dimensional representation of the segmentation map. This process, further detailed in [8], allows the acquisition system to provide interventional assistance to the neurosurgeons performing tumor resection through an immersive exploration of the scene.

**Figure 2:**
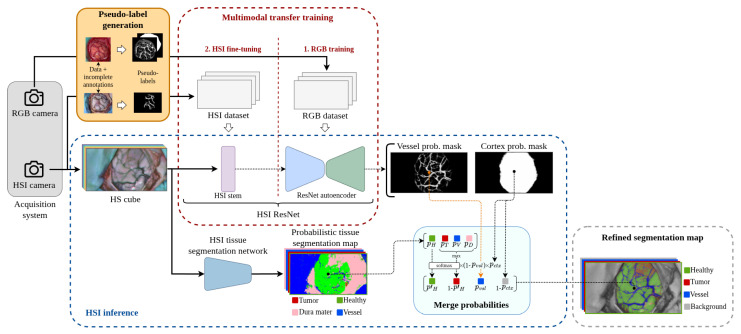
Block diagram illustrating the proposed strategy for improving brain tumor detection by refining the output of any HS tissue segmentator. To address the absence of a complete ground truth in both RGB and HSI domains, the block *Pseudo-label generation* extracts vascular and cortical annotations (Section 3.2) that enable the *Multimodal transfer training* process (Section 3.4). Through 2 steps, the *HSI ResNet* (Section 3.3) is first pre-trained using the RGB dataset to adjust the *ResNet autoencoder*. Then, the *HSI stem* is fitted with the HSI dataset, obtaining a model capable of segmenting the brain surface and its blood vessels of a given HS image. These segmentation masks are subsequently used during the *HSI inference* phase to be combined with the probabilistic output of the segmentation network in the *Merge probabilities* block (Section 3.5) to achieve the *Refined segmentation map*.

In this study, one of the primary objectives is to explore the utilization of the collected RGB information to test its capability to enhance HS-based tissue segmentation. Following the diagram depicted in Figure 2, the main element that articulates the adaptation between the RGB and HSI domains is illustrated as the *Pseudo-label generation* module. There, the higher resolution of RGB images is exploited to create a dataset in which the lack of complete medical annotations is compensated through the generation of cortical and vascular pseudo-labels. With these pseudo-labels, as represented in the *1. RGB training* step inside the *Multimodal transfer training* block, it is possible to train a robust model in the RGB domain (*ResNet autoencoder*) that captures morphological patterns present in the parenchyma. This model can then be transferred into the HS domain to perform the desired segmentation using the so-called *HSI ResNet*. The transfer between image modalities is enhanced in the *2. HSI fine-tuning* step by fitting the *HSI stem* with the HS dataset to adapt the larger number of spectral characteristics to the RGB pre-trained *ResNet autoencoder*. Inside the *2. HSI fine-tuning* step, the sparsely annotated ground truth provided by neurosurgeons is supplemented with blood vessel pseudo-labels generated to accomplish, in this manner, a reliable segmentation of the brain surface and its vascular structures.

The fusion of cortical and tissue segmentation probabilities depicted in the *Merge probabilities* block in Figure 2 represents the final stage of the procedure developed. Based on observations reported in [7], the tissue segmentation map is assumed to be possibly flawed in the distinction between tumor, blood vessels, and dura mater. Consequently, complete confidence is placed on the cortical and vascular detections produced by the *HSI ResNet*. Once both cortical segmentation masks are applied to the tissue segmentation map, the only pixels left to be considered as potential tumor samples are the ones within the cortex mask that are not overlapped by the vessel mask. For these remaining samples, the higher sensitivity for detecting healthy pixels shown by all algorithms tested in [7] justifies prioritizing the tissue segmentator criterion for pixels identified as healthy. Consequently, the rest of the pixels that are not classified as healthy are, by exclusion, reconsidered and assigned to the tumor class.

Although validation experiments are conducted using multiclass segmentation networks selected from the literature, the suggested strategy reduces the demands on the HS segmentation network, effectively transforming the problem into a de facto binary classification in which a precise distinction between tumor, vascular, and dura mater classes is no longer necessary. This approach, therefore, requires the HS segmentator to distinguish only between healthy tissue and other types, while still yielding a final multiclass segmentation map.

Given the challenges addressed in this work, the main contributions can be summarized as follows:

- A strategy for correcting non-healthy misclassified pixels is introduced, showing its effectiveness for improving the tumor detection capability of any given segmentator in the HS domain.
- To propose an RGB and HSI multimodal training methodology based on incomplete annotations capable of producing an accurate segmentation of the brain parenchyma and its blood vessels.
- To suggest the complementation of an HSI–NIR image source with an RGB image modality as a factor of improvement for brain tumor detection by enabling the proposed misclassification error correction strategy.

## 2. Related Work

### 2.1. Hyperspectral Imaging in Brain Tumor Detection

The application of HSI technology to in vivo brain tumor detection is a relatively recent development, with significant contributions coming from the HELICoiD project [9], where the application of ML techniques was explored to obtain representation maps capable of discriminating healthy from tumor pixels. The ideas applied to [9] were continued through the NEMESIS-3D-CM project [10] with a more extensive investigation and collection of HS material applied to intraoperative tumor diagnosis. As a result, a multimodal database composed of HS, but also RGB and depth information, from 193 patients was compiled under the name of SLIMBRAIN database [8]. The available ground truth in [8,9] consisted of sparse annotations indicating four main classes: healthy tissue, tumor tissue, blood vessels and background. With this material, several ML and neural-network-based approaches were tested on the tumor detection task. A relevant sample of them can be found in the benchmark performed by Leon et al. [6], where the use of convolutional neural networks (CNNs) [11] of one and two dimensions obtained competent results [12].

### 2.2. In Vivo Brain Cortex Segmentation

The literature addressing the delineation and segmentation of the cerebral cortex is extensive in the context of magnetic resonance imaging (MRI); however, research focusing on in vivo operations remains comparatively limited. The only examples of cerebral surface segmentation that the authors of this work are aware of are found in [13,14]. Whereas Luo et al. suggested in [13] a methodology to segment surgical instruments and relevant tissues for surgical guidance, Fabelo et al. performed, in [14], a delineation of the exposed brain cortex after craniotomy during in vivo brain tumor operations with the intention of removing possible sources of error in the subsequent tumor detection task. Although the work of Fabelo et al. shares goals similar to those exposed in this research, both [13,14] approaches rely on fully annotated cortical tissue regions, in contrast to the strategy proposed in this work.

### 2.3. Cortical Blood Vessel Segmentation

As in the topic of in vivo brain surface segmentation, most publications dealing with the identification and mapping of cortical blood vessels are based on datasets from MRI or CAT scans [15]. In this kind of modality, a large number of specialized deep learning solutions can be found, such as the DeepVesselNet architecture introduced by Tetteh et al. [16]. However, within an imaging modality similar to the one used in the present study, a significant number of papers on retinal vessel segmentation using RGB imaging can be found, in which both DL-based approaches [17] and algorithmic methods are explored. One of the most popular examples of vessel detection algorithms that is not based on DL solutions is the Frangi vesselness filtering [18,19]. Despite its proven goodness, the applicability to the problem addressed in this paper is limited due to the greater variability in thickness between the brain cortical vessels and the angiography images, the kind of captures where the Frangi filtering is applied primarily. Another relevant publication concerning the research conducted in this paper is the solution based on linear operators proposed by Ricci and Perfetti [20].

In terms of purely in vivo brain blood vessel segmentation, there are two publications directly addressing this matter. In the first one, Haouchine et al. [21] provided a strategy that extends the available human craniotomy dataset using neural style transfer, thereby enhancing the generalization capability of the trained model. On the other hand, Wu et al. [22] studied the segmentation of vascular structures using wide-field optical microscopic images to help study the oxygenation of different areas in the brain of mice.

### 2.4. Limited Supervision in Medical Image Segmentation

Thus far, most of the referenced publications rely on fully annotated datasets. However, a common problem that arises in medical image segmentation is the lack of a complete ground truth that forces the application of alternative procedures to maximize the utility of available annotations. In [4], Tajbakhsh, Jeyaseelan, and Li et al. established a taxonomy of the casuistry of the problem depending on the degree of incompleteness of the annotations and the approach applied to overcome it. Regarding the purpose of the methodology presented in this work, the embedding similarity procedure developed by Huang et al. in [23] is of great significance. There, a lymphoma segmentation in positron emission tomography (PET) images faces the absence of complete annotations in part of the dataset. To enhance the tumor detection capacity of the network employed, the features extracted from the last layers are used in a loss function that enforces the tumor embeddings to be close to each other but far from non-tumor-generated features in terms of cosine similarity. A different method applied, this time to brain tumor segmentation in MRI with only image-level annotations, is described in [24] by Patel and Dolz. The equivariance constraint, whereby the class activation map (CAM) outputted by a neural network should be the same for an image and its affine-transformed version, was exploited to ensure the spatial consistency of the segmented result.

### 2.5. Pseudo-Label-Based Supervision

Complementary to the regularization constraints imposed to the neural network output when the used dataset contains weak or incomplete annotations, there is a commonly exploited approach based on pseudo-labels. Pseudo-labels are annotations, normally generated through automatic procedures, that capture the majority of elements to be detected by the neural network being used, but, due to their unsupervised origin, are prone to be flawed. However, if the technique employed to extract them is robust enough, it allows an initial learning point for the model. As presented by Luo et al. [25], these pseudo-labels can be generated by a neural network under scarce supervision, scribbles for cardiac MRI segmentation in this case, by mixing the output of two different decoders sharing the same encoder. Another approach discussed by Zhang et al. in [26] consists of generating a prior set of pseudo-labels that represent vascular structures in X-ray angiograms. These pseudo-labels are later refined based on the model uncertainty estimated over its vessel predictions. Pseudo-labels may also result from simplified annotation strategies designed to reduce labeling time and complexity. The Vessel-CAPTCHA method proposed by Dang et al. [27] generates vessel pseudo-labels from brain angiograms using a CAPTCHA-inspired approach, in which manual intervention is limited to selecting grid regions containing vascular structures. Then, image processing techniques extract vessel contours to form the pseudo-label masks.

### 2.6. Multimodal Learning for Medical Image Segmentation

Another alternative for producing robust models in the medical image segmentation field is to incorporate multiple annotated datasets coming from different image modalities to exploit their common features. This is exemplified in [28], where the characteristics extracted from brain blood vessels captured in an angiography are transferred into the venography domain with a limited set of annotations. The CS-CADA method developed by Gu et al. [29] also illustrates how the multimodal learning strategy is applied to achieve robust segmentation in a target domain with scarce annotations applying domain-specific batch normalization to better address the heterogeneity of the modalities. Another case for brain vessel segmentation across different domains is proposed in [30]. There, various domains containing images with vessels or similar contours are condensed under an image-to-graph methodology.

The multidomain learning idea can also be implemented in a collaborative way under the concept of federated learning, in which multiple decentralized participants provide different sources of images with heterogeneous modality, building a common model. An example of this approach can be found in the framework proposed by Galati et al. [31], where the commonly built model can be transferred and adapted to the target domain of each participant. Other cases of models trained with extensive collections of heterogeneous datasets are UniverSeg [32] and MedSAM [33]. Both of them aim at achieving reliable segmentation regardless of the target domain.

Concerning the adaptation of the RGB image domain into the HS for medical imaging, the literature is limited. The benefits of combining RGB and HS imaging modalities are mostly assessed in fields such as remote sensing or microscopy imaging. In the remote sensing domain, Yuan et al. [34] addressed the super-resolution problem in HSI by pre-training a CNN model on a low- to high-resolution RGB dataset that is then transferred to the HS modality. For microscopy applications, Ye et al. proposed a hybrid training between the RGB and HSI domains for differentiation between live and dead cells in microscopy images in [35].

## 3. Materials and Methods

The following sections describe the way both RGB and HSI images are captured, pre-processed, and how their partial annotations are obtained (Section 3.1). This is followed by a detailed explanation of how these partial annotations are used to generate the pseudo-labels (Section 3.2) and how these pseudo-labels are used in the multimodal training process of the neural network employed (Section 3.3) to achieve a reliable segmentation of the brain surface and its blood vessels (Section 3.4). Finally, the probability combination between the segmentation masks and the HS segmentation map is depicted in Section 3.5.

### 3.1. Data Acquisition

The HS images used in this work are a selection of 67 different patients undergoing brain surgery extracted from the SLIMBRAIN database [8]. All images were captured at the University Hospital 12 de Octubre in Madrid (Spain). The study was carried out following the Declaration of Helsinki guidelines and was approved by the Research Ethics Committee of the Hospital Universitario 12 de Octubre, Madrid, Spain (protocol code 19/158, 28 May 2019).

#### 3.1.1. Acquisition Systems

The central element used for collecting the biological data, and the only source of hyperspectral images used in this work, is a snapshot hyperspectral camera (Ximea GmbH, Münster, Germany). It is a first-generation MQ022HG-IM-SM5X5 with a sensor resolution of 2045 × 1085 pixels, capable of capturing 25 bands ranging from 665 nm to 960 nm. The 25 spectral filters are arranged in a 5 × 5 mosaic pattern repeated throughout the sensor; therefore, the resulting hyperspectral cube has a spatial resolution of 409 × 217 pixels with 25 spectral bands for each one of them.

Over a period of almost five years, the capturing system has undergone four major changes in its composition and operability. Each of these four upgrades is detailed in [8], where the corresponding acquisition system is referred to as *SLIMBRAIN* prototype 1, 2, 3 or 4 depending on its version. In this work, all images used were obtained using versions 1, 2 and 3.

**Figure 3:**
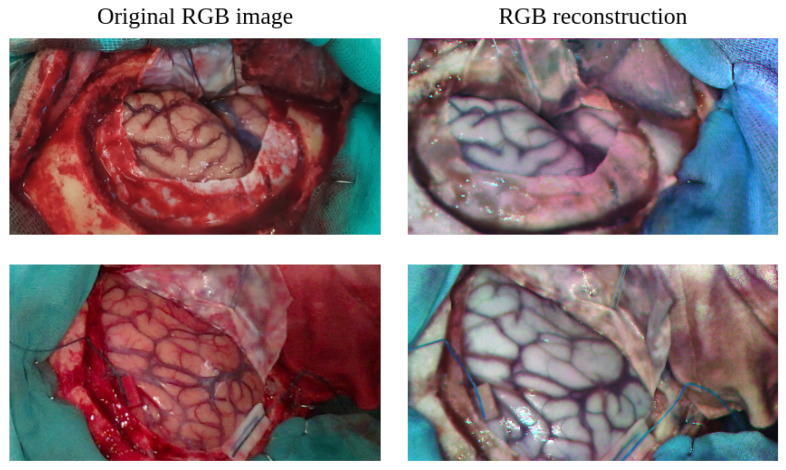
Comparison between the cropped images taken with an RGB camera (left column) and the RGB reconstruction from the hyperspectral cube (right column).

In *SLIMBRAIN* prototype 1, the HS snapshot camera was mounted on a tripod alongside two gooseneck optic fibers that redirected the illumination provided by a 150 W halogen bulb (Dolan-Jenner, Boxborough, MA, USA). From *SLIMBRAIN* prototype 2 onward, the snapshot HS camera and optical fibers are fixed to a crossbar that holds a Zaber Technologies linear stage for better manipulation. The RGB acquisition for versions 1 and 2 relied on regular smartphone cameras from heterogeneous models. In *SLIMBRAIN* prototype 3, depicted in Figure 1, the camera and lighting handling remain the same, but the Dolan–Jenner bulb is replaced by another 150 W bulb from Osram GmbH, Münich, Germany. In this version, an Intel L515 LiDAR with an RGB sensor with resolution of 1929×1080 and 8 bits per color channel is also included in the linear stage and is set as the default RGB collecting method. It is important to note that the HS linescan camera shown in Figure 3 is not involved in the proposed solution.

#### 3.1.2. Capturing Procedure

As previously described, the dataset used in this work comes entirely from the SLIMBRAIN database [8] which is composed of intraoperative multimodal images taken at the Hospital Universitario 12 de Octubre, Madrid, Spain. These interventions consisted mainly of resections of different types of brain tumors such as astrocytoma, meningiomas, and brain metastases. Surgical interventions for non-tumor pathologies such as aneurysms and arteriovenous malformations were also captured.

All images were acquired after the craniotomy was performed, particularly once the dura mater was removed, exposing the brain cortex. For some patients, the cortical area was also captured after the tumor resection was carried out.

At the time of capture, the acquisition system is placed in a working distance range that goes from 21 to 50 cm, measuring it with any of the ranging devices depending on the system version: a laser rangefinder for *SLIMBRAIN* prototypes 1 and *2*, and the LiDAR of the L515 device for *SLIMBRAIN* prototype 3. The chosen working distance ensures a safe proximity range for the patient whilst allowing a good fit of the craniotomy in the hyperspectral images.

Once the system is properly placed, the hyperspectral image is taken along with the RGB capture. In version 3 of the acquisition system, the L515 LiDAR retrieves the RGB and the distance images at the same time, whereas prior to version 3, the RGB image was obtained with a regular mobile camera.

#### 3.1.3. Data Pre-Processing

In order to convert radiance to reflectance and also mitigate the effect of the sensor noise, the different arrangement of the gooseneck fiber optics and the various lighting bulbs used throughout the versions of the acquisition system, the hyperspectral cubes are calibrated using a white reference cube of a polymer with nearly ideal Lambertian properties and a dark reference cube. For each version of the acquisition system, a set of captures of the white polymer (SphereOptics 461 GmbH, Herrsching am Ammersee, Germany) are taken covering a distance range of 30 to 70 cm every 5 cm and angles from 30 to 80 every 10 degrees. This set of white references allows compensation for the variations in distance and angle of capture between different hyperspectral images.

The snapshot camera manufacturer warns about cross-talk between adjacent pixels inside each mosaic pattern of the sensor filters [36]. To amend this effect, after applying the black and white calibration, each spectral signature must be multiplied by a spectral correction matrix provided by camera sensor manufacturer IMEC, Leuven, Belgium.

#### 3.1.4. RGB Image Reconstruction from HSI

For visualization purposes, it is desirable to have a method to reconstruct an RGB image from a hyperspectral cube in such a way that the color components resemble as closely as possible the real appearance of the scene, obtaining what can be called a pseudo-RGB image (pRGB). However, as the snapshot camera is centered in the near-infrared region of the spectrum, it is only able to detect the red component in its fourth band (712.4 nm). For reconstructing green and blue components, which are assumed to be in the range 495–570 nm and 450–495 nm, respectively, their closest multiples are selected from the bands captured by the camera. The green component is coarsely approximated by the 23rd band (940.9 nm) because its second harmonic is located at 470.5 nm, falling almost at the transition between green and blue. The blue component is associated with the 20th band (913.7 nm) because its second harmonic (456.9 nm) is in the blue color range.

As neither the lighting source nor the camera sensor has flat spectral response, it is necessary to compensate for their responses so that the reconstructed RGB components are balanced. This can be achieved by selecting the 4th, 23rd, and 20th bands after applying the white reference calibration described in Section 3.1.3. The calibrated RGB components are then equalized independently and the contrast of the overall image is adjusted, providing the result shown in Figure 3.

#### 3.1.5. Hyperspectral Image Labeling Procedure

Once the hyperspectral images have been captured and the intervention has been completed, the neurosurgeon in charge of the operation can proceed with the labeling of the image. The tissues considered to be relevant and, therefore, annotated are dura mater, cortical blood vessels, tumor tissue (if applicable), and healthy tissue. Healthy tissue is taken to be those regions that the neurosurgeon is highly confident in not being affected by any pathology. Tumoral tissue, on the other hand, is delimited according to the neurosurgeon’s criteria acquired during the tumor resection. Decisions on labeling of both healthy and tumor tissue are complemented by pathologist information on biopsies taken during the operation.

**Figure 4:**
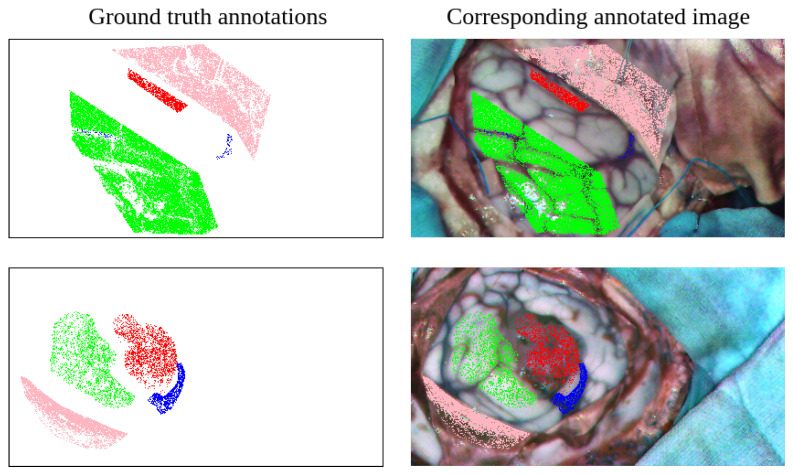
Ground truth annotated by the neurosurgeon (left column) displaying tumor pixels in red, healthy tissue in green, blood vessels in blue, and dura mater in pink color. In the (right column), the same ground truth annotations are overlaid on the RGB reconstruction from the corresponding HS images.

To perform the annotation, the neurosurgeon uses a semi-automatic labeling tool via a graphical user interface, used in previous hyperspectral brain imaging works [8,37]. The labeling procedure consists of the surgeon selecting a reference pixel whose class can be reliably determined. Following this, to mark more pixels belonging to the same class, a threshold is adjusted based on the spectral angle mapper (SAM) [38]. Once the threshold is set so that a relevant number of pixels can be selected, a polygon is drawn, enclosing the samples belonging to the class to be labeled. Thus, samples from other classes that are within the similarity threshold are not included. This process attempts to balance the generation of reliable ground truth by requiring the neurosurgeon to devote a reasonable amount of time to the process. As a result, a sparse annotated dataset is generated in the form shown in Figure 4.

#### 3.1.6. Dataset Composition

From the complete collection of captures obtained throughout the five years of data collection, data from 67 patients were selected. The rest of the images were not included because of inadequate capture conditions, such as low lighting of the scene or blurring of the image. Patients whose craniotomy was too small or undergoing a severe second brain surgery were also discarded due to the lack of clear contours. In addition, HS images without their corresponding RGB image were not considered for this work.

Of the 67 selected patients, 50 of them had a cancer-related condition, whether it originated in the brain or was produced by metastasis, but only 31 had visible tumor tissue on the brain surface. The rest of them were affected by cerebrovascular diseases. As a result, the total number of labeled pixels is shown in Table 1. The imbalance between the four classes is especially noticeable when comparing the ratio between healthy and vascular samples, where there are almost ten times more annotated pixels of healthy tissue than of vascular tissue.

#### 3.1.7. RGB Simplified Annotations

As RGB images are not considered to contain relevant information for tumor detection, neurosurgeons do not take the time to label them. However, the higher resolution of the RGB images with respect to the HS images provides a complementary source of morphological information that, given the motivation of this work, may be useful for the task of segmenting the blood vessels and delimiting the boundaries of the brain surface.

**Figure 5:**
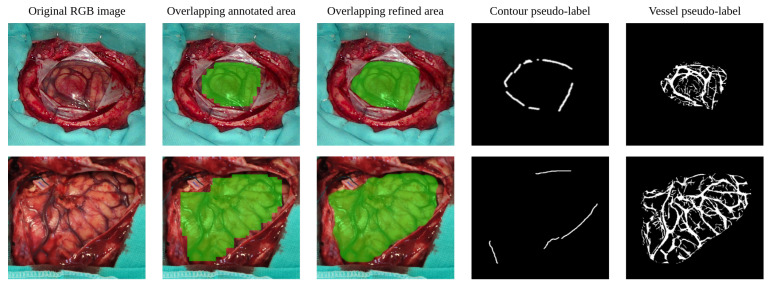
Details of the captures taken with the L515 LiDAR RGB camera (first column), the patch-based manual annotations (second column) and pseudo-labels obtained for the RGB captures (third column) along with the contour and vessel pseudo-labels (fourth and fifth columns).

To help extract this information in a simplified manner, a rapid manual labeling procedure is applied. In this procedure, where no medical training is required, the exposed brain surface region is marked down through a set of adjustable size square patches. The goal is to cover as much of the brain surface as possible, while avoiding including pixels from any region other than the brain surface. Once the annotation process is completed, the patch collection is combined to create a mask that coarsely outlines the shape of the exposed cortex, as can be seen in the second column starting from the left in Figure 5.

The mask obtained forms base annotations that contain positive examples of cerebral tissue. To automatically mark negative examples, non-overlapping square patches are sampled from the area not covered by the positive mask. In order to have sufficient confidence in not including brain tissue pixels as negative samples, a safety margin is set between the positive mask and the negative sampling area. The size of the negative patches is fixed at 217 pixels, corresponding to the height of the HS images, while the safety margin is set at ⌊217/2⌋=108 pixels.

### 3.2. Pseudo-Label Generation

The performance of the model in segmenting the brain surface and its blood vessels on HS images is highly dependent on the RGB training procedure and, to a lesser extent, on the HSI fine-tuning of the model. These two training steps are only made possible by the generation and refining of the pseudo-labels detailed in the following sections. More specifically, Section 3.2.1 and Section 3.2.2 cover both the estimation of the cortical surface and the extraction of relevant boundary contours, respectively, using the RGB dataset. Section 3.2.3 details the process of generating blood vessel pseudo-labels for both RGB and HSI domains. Finally, Section 3.2.4 explains the pre-processing performed on the HS dataset ground truth.

#### 3.2.1. RGB Brain Cortex Annotation Refining

To complete the manually labeled regions described in Section 3.1.7, the method detailed in this section has the primary objective of extending the boundaries of the annotations as close as possible to the actual boundaries of the exposed brain surface. As this refinement method is intended to be fully automatic, it must be robust enough to provide a reliable approximation of the shape of the cortex. For this reason, the proposed method makes basic assumptions about the morphological and chromatic composition of the RGB captures. First, it is assumes that there can only be two types of tissue in the patch-based annotations: cortical tissue (either healthy or tumor) and blood vessels, and that cortical tissue is expected to appear lighter than vascular tissue. Therefore, pixels that have color and brightness similar to the original annotations and are also close to them are likely to be of the same type. Following this principle, the originally annotated mask can propagate through pixels similar to those taken as blood vessels in the labeled region. Due to the high vascularity of brain tissue, propagation using blood vessels makes it easier to cover the cortical surface. Then, the areas surrounding the newly considered vessel pixels can be compared to the brain surface in the manually marked area to decide whether they are also cortical tissue. To facilitate the comparison between the pixels in the annotated mask and the rest of the image, the image is divided into zones of similar pixels using the K-means algorithm [39]. The particular algorithmic treatment of these clusters is described in detail in Algorithm S1 in the Supplementary Materials. The result of the refining method is shown in the third column starting from the left in Figure 5.

#### 3.2.2. RGB Brain Surface Perimeter Approximation

Once the manually annotated region is extended and refined closer to the surface boundaries of the cerebral cortex, the edges that form the boundaries of the brain parenchyma can be extracted. These contours contain relevant information that can be used during model training to apply regularization cues for better delineation of the boundaries of the cerebral cortex. In order to extract them, the Canny algorithm is used, filtering the smaller detected edges that might not be part of the brain surface bounds. Then, the edges close to the refined mask are searched in the area between the 5% eroded version of the perimeter of the refined mask and its 10% dilated version. To cover this area faster, the image is divided into superpixels using the SLIC algorithm [40], expanding the limits of the eroded mask from superpixel to superpixel until any of its boundaries meets an edge or until the dilated limit is exceeded. As a result, the strongest and closest contours to the refined mask can be identified, producing the output that can be seen in Figure 5 (fourth column starting from the left).

#### 3.2.3. Cortical Vessel Pseudo-Label Generation

Although the generation of pseudo-labels representing desirable targets that the model must learn is applied to both RGB and HSI domains, this generation process is focused on HS images. This is because it is the only image source that contains cortical annotations and, therefore, the only modality where objective evaluation metrics can be used. The pseudo-label generation approach is an application of the work presented in [20] based on the work proposed by [41].

In [20], the process that obtains pseudo-labels from the HS images works under the assumption that vascular tissue tends to be darker than the rest of the cerebral cortex. Therefore, the inverted grayscale image taken from a given band can be used to capture the intensity of the blood vessels. Given the elongated morphology that vascular structures normally exhibit, this intensity can be extracted by multiplying the inverted grayscale image by a so-called linear operator. This linear operator is formed by a set of *N* squared kernels with a dimension of k×k pixels, which are all zeros except for a single straight line running from side to side through the center. The methodology detailed in [20], uses two linear operators, both of them with N=12 kernels covering 12 different orientations (each line is progressively offset by 15 degrees, as Figure 1 from [20] illustrates). In order to capture as much of the range of sizes that blood vessels can exhibit, the two linear operators use kernels with different dimensions *k*: one to detect thinner structures like capillaries, and another for thicker contours such as veins and arteries. The working mechanism of the linear operators and their parametrization are explained in Section S2 of the Supplementary Materials.

The generation of blood vessel pseudo-labels for the RGB dataset is performed through the same method described above for the HS images, with the exception of certain parameters whose value is adapted to the RGB dataset, as described in Section S2. The resulting vascular contours extracted by the linear operator method are shown in Figure 5, fifth column from the left.

#### 3.2.4. HSI Ground Truth Densification and Background Complementation

In view of the scarcity and sparsity of the HSI ground truth, a simple way to increase the number of labeled pixels without the supervision of a neurosurgeon and minimizing the possibility of mislabeling any pixels is to perform a closing morphological operation on the labeled samples. Both dilation and erosion operations are performed using an 11×11 elliptical kernel, a size large enough to ensure that no annotated pixel is left isolated. The result of this closure operation is an incomplete but densified version of the HS ground truth. The refinement process described in Section 3.2.1, designed to complete small areas between given annotations and the boundaries of the brain parenchyma, cannot be applied to the HS dataset, as the densified ground truth does not cover enough surface area of the cerebral cortex in most cases.

In addition, as the only labeled part in the HS images that does not belong to the brain surface is the dura mater, there is a significant label imbalance for a task aimed at segmenting the brain cortex. In order to generate background samples that reliably do not belong to the cortical area, the same clustering strategy used in the RGB brain cortex label refining (Section 3.2.1) is applied. In this case, after following the steps described in Section S3 of the Supplementary Materials, the eight most dissimilar clusters to the brain surface labeled samples are selected to be integrated into the background mask.

### 3.3. Neural Network Architecture

**Figure 6:**
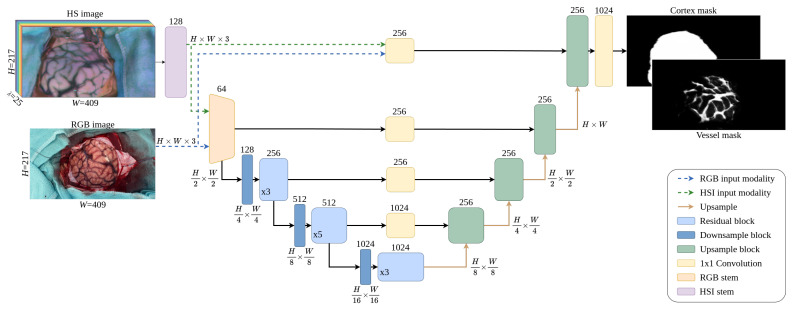
Architecture of the neural network *HSI ResNet* used for segmenting the brain surface and the cortical blood vessels. Green and blue dashed arrows show the two image modalities that the network can process. The number placed above each diagram block indicates its feature dimension.

The neural network used in this work, depicted in Figure 6, is designed considering the empirical basis set by S. Seidlitz, J. Sellner, J. Odenthal et al. in [5], where it is concluded that an encoder–decoder type of NN architecture offers the best performance for organ segmentation in HS images, particularly when the entire HS image is fed to the network instead of partitioning the input into patches. The proposed architecture is designed to work with two possible image source modalities, one at a time: RGB and HSI. The structure is conceived primarily to process HS images, but also to handle RGB captures to extract relevant information when training with them. When the network operates in RGB mode, the image is fed directly to the *RGB stem* (orange block in Figure 6) which is implemented following the so-called ResNet-C structure described in [42]. When the network processes an HS image, it is first passed through the *HSI stem* (purple block in Figure 6), which is formed by three valid 2D convolutional layers, each followed by a batch normalization layer [43] and a leaky ReLU activation [44]. The main purpose of the *HSI stem* is to reduce the spectral dimensionality of the HS cube and to adapt it so that it can be fed to the *RGB stem*.

The rest of the network follows an encoder–decoder architecture that uses a lightweight ResNet [45] implementation as its backbone, building the encoder with 14 residual blocks grouped in three stages according to its working resolution. The downsample block (dark blue block in Figure 6) is based on the ResNet-D structure, and the residual block (light blue block in Figure 6) follows the ResNet-B implementation, both following the structures described in [42].

The decoder performs the upscaling of the embedding produced by the encoder, incorporating skip connections with each stage of the encoder. In each stage of the decoder, the output of the previous block is upsampled by a factor of 2 using the nearest-neighbor interpolation. The encoder output with the same resolution is then linearly projected and added to the upscaled output. Then, inside each of the green blocks in Figure 6, two 3 × 3 convolutions are applied, followed by a batch normalization layer and leaky ReLU.

The output of the decoder is passed to a feedforward network resulting in two binary images with the same spatial resolution as the input, each of them corresponding to the segmented brain surface and the cortical blood vessels, respectively. As both of the output images, marked as *Cortex mask* and *Vessel mask*, do not represent mutually exclusive classes, the sigmoid function is used in the final activation, providing a probabilistic result.

### 3.4. Multimodal Training Methodology

**Figure 7:**
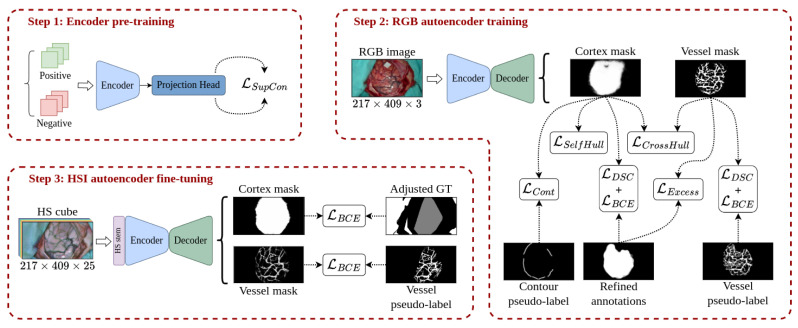
Overview of the multimodal training methodology used in this work based on three steps: 1. Encoder pre-training by supervised contrastive loss based on positive and negative examples in the form of RGB patches from the parenchymal area and its surroundings. 2. RGB autoencoder training using multiple supervision signals based on the generated psuedo-labels. 3. Autoencoder fine-tuning and *HSI stem* fitting using the pre-processed ground truth and the blood vessel pseudo-labels.

The proposed training pipeline depicted in Figure 7 aims to compensate for the lack of a fully annotated dataset in the HS domain, the target modality, making use of the information extracted from a different imaging modality, the RGB domain, so that a complete segmentation of the cortical vessels and the exposed brain surface can be performed.

The different parts of the neural network described in Section 3.3 are trained in the order shown in Figure 7. It starts with the pre-training of the encoder in a supervised contrastive fashion following the work proposed in [46], using the annotated patches extracted as indicated in Section 3.1.7. Next, the RGB dataset composed of the RGB captures and the pseudo-labels generated as shown in Section 3.2 is used to train both the encoder and decoder parts of the network. The last step consists of training the so-called *HS stem* along with the fine-tunning of the encoder and the decoder, using for this purpose the densified version of the ground truth and the generated blood vessel pseudo-labels.

#### 3.4.1. Encoder Pre-Training

The purpose of this stage is to facilitate the training of the autoencoder in the subsequent two stages by enabling the encoder to distinguish between patches of cortical tissue and patches taken from the craniotomy surroundings. To train the encoder to differentiate between the two types of image, the dataset is divided into positive examples (images of the brain surface) and negative examples (images from other regions). Positive examples are composed of manually labeled patches detailed in Section 3.1.7, while negative examples come from patches automatically extracted from the surroundings of the annotated region.

The encoder pre-training is performed following the supervised contrastive learning strategy proposed by Khosla et al. [47]. In order to do so, the pooled and normalized output of the encoder is passed to a projection network referred to as the *Projection Head* in Figure 7, Step 1. This projection network, which is discarded once the encoder pre-training is finished, is composed of two linear layers with an embedding dimension of 256. The output of the projection network is used to calculate the supervised contrastive loss, as formulated in Equation (2) of [46] (https://github.com/HobbitLong/SupContrast/blob/master/losses.py, accessed on 14 May 2025 ) and indicated as $L_{SupCon}$ in Figure 7, Step 1.

Both the positive and negative examples used in this process are rescaled to the [0,1] range and subjected to a variety of random data augmentations. Particularly, as suggested in [47], a combination of morphological transformations such as random cropping, color distortion, and Gaussian blurring is applied. The commonly used random cropping is omitted given the patch-based nature of the data used in this training stage, which makes it an already cropped version of the brain parenchyma to be detected.

#### 3.4.2. RGB Domain Training

The next step consists of training the encoder and decoder parts with the RGB dataset. This stage can be considered the main part of the whole training methodology, as it is the point at which the model receives the most significant inputs that will condition its performance in the final cortical and vascular segmentation task on the HS dataset. To ensure the transferability of the model from the RGB domain to the HSI modality, the RGB dataset generated in Section 3.1.6 is adapted to be as similar as possible to the HSI dataset in terms of the aspect ratio of the captures and the size of the craniotomy relative to the size of the image. As the labeled cortical tissue (healthy, tumor and vascular tissue) in the HS ground truth occupies, on average, 13.2±8.1% of the image surface, the high resolution and wide field of view of the RGB images can be exploited so that they are cropped to make the refined annotated mask described in Section 3.2.1 to take up to 20% of the image surface while maintaining the same aspect ratio as the 217×409 resolution of the HS cubes. Then, the dataset is resized to the 217×409 resolution and its values are rescaled to be within the [0,1] range. The data augmentation procedure of [47] is also followed in this stage using a combination of random cropping, color jittering, and Gaussian blurring along with random flips.

As Figure 7, Step 2 shows, the calculation of the loss for the cortical and vascular segmentation masks is integrated using multiple terms. The main element of the loss function can be considered to be the computing of the binary cross-entropy (BCE) plus the complement of the Dice similarity coefficient (DSC) [48] of the cortex mask with respect to the refined annotations and between the vessel mask with the vessel pseudo-labels, both expressed as $L_{DSC}+L_{BCE}$. The rest of the loss elements work as regularization terms responsible for enhancing certain aspects of the learning process:

- $L_{Cont}$: Similar to the technique presented in [49], the *contour loss* $L_{Cont}$ aims to guide the limits of the cortex segmentation mask so that it matches the boundaries of the brain surface produced in Section 3.2.1. To do so, the limits of the cortex mask generated by the network are extracted using the Canny algorithm and dilated with an 3×3 elliptical kernel. Then, the BCE is calculated between the extracted edges and the contour pseudo-labels only for the pixels where the contour pseudo-labels are greater than zero. Thus, the $L_{Cont}$ term penalizes the predicted cortical mask when it is not adjusting to the contour pseudo-labels.
- $L_{SelfHull}$: As the exposed cerebral cortex is integrated by a single region, the segmentation mask of the cortex cannot be composed of multiple unconnected areas. If this occurs, it might be indicative that the model has partially detected the cortical area or has marked elements that do not belong to it. To force the generation of a single solid mask, the *self-hull loss* term $L_{SelfHull}$ computes the complement of the DSC between the predicted cortex mask and the area enclosed by its own concave hull [50] (https://pypi.org/project/concave-hull/0.0.1/, accessed on 14 May 2025), following a similar idea to the one suggested by Guo et al. in [51]. Hence, sparse segmentation of the cortical area or scattered activations in external zones produce empty areas within the concave hull of the predicted mask, leading to high loss values. It is important to note that this term must be used in conjunction with the $L_{DSC}+L_{BCE}$ constraint to avoid the cortex mask to adjust to a bad concave hull perimeter.
- $L_{CrossHull}$: In a similar manner to *self-hull loss*, the segmentation mask for blood vessels cannot be active outside the bounds of the predicted brain cortex mask and, on the other hand, the brain cortex mask should be confined within the limits of the detected vessels. Hence, the perimeters of both masks should be as close as possible. To constrain the consistency between both network outputs, the *cross-hull loss* calculates the complement of the DSC between the areas contained within the concave hulls of the predicted cortex and vessel masks.
- $L_{Excess}$: Complementary to the $L_{DSC}+L_{BCE}$ element applied to the blood vessel segmentation, the *excess loss* term penalizes the activation of the predicted blood vessel mask outside the bounds of the refined annotations. This penalization is implemented as a minimization of the overlapping between the predicted vessel mask and the complement of the vessel refined annotations through the following equation:
  (1) $$L_{Excess}=\frac{1}{B}\sum_{b=1}^{B}(\log(\alpha \cdot \mathrm{DSC}\left(1-Y_{b},\hat{Y_{b}}\right)+1))$$
  where *DSC* represents the Dice similarity coefficient between the complement of the refined annotation 1−Y and the predicted vessel mask $\hat{Y}$ for the $b^{th}$ image inside a batch with size *B*. The factor α is set to 10 for greater penalization when DSC is close to one, whilst the logarithmic function smooths the slope of the loss function.

The complete loss function is calculated as follows:

(2) $$\begin{array}{c} L=L_{ctx}+L_{SelfHull}+L_{Cont}+L_{vsl}+L_{CrossHull}+L_{Excess} \end{array}$$

#### 3.4.3. HSI Model Fine-Tuning

The last stage of the training procedure consists of the adaptation of the model trained in the RGB modality to the HSI domain. The available sources of supervision that can be applied at this stage are the blood vessel pseudo-labels generated as described in Section 3.2.3 and the densified ground truth of Section 3.2.4. The multiclass composition of the densified ground truth is of limited use at this stage of the training; therefore, it is transformed and redefined to have only three classes: inner, which is the area contained by the concave hull of healthy, tumor, and vascular samples; outer, the junction of dura mater samples with the background labels generated in Section 3.2.4; and unknown, the remaining unlabeled pixels.

As depicted in Figure 7, Step 3, the fine-tuning of the model is performed by calculating the partial BCE loss between the predicted cortex mask and the adjusted densified ground truth, but only on pixels belonging to the inner and outer classes. Thus, the loss function expects the predicted cortex mask to be active in the inner class but inactive in the outer class.

For the predicted blood vessel mask, only the vessel pseudo-labels that are within the inner label are considered for calculating the BCE loss between them and the blood vessel prediction. The regions belonging to the outer class are taken into account for the loss calculation by penalizing any activation produced inside them, as it would be caused by a wrong detection of vascular tissue outside the brain surface.

At this stage of training, all parts of the model except the *HS stem* are adjusted for the segmentation of the brain cortical area and its blood vessels. Hence, the learning rate applied to all layers except the HS stem is divided by a factor of $10^{3}$, to ensure minimal adaptation of the weights of each layer, but to allow the running mean and variance inside the batch normalization layers to adjust to the HS data.

### 3.5. HS Image Combined Inference

As Figure 2 indicates, the final output map of the proposed pipeline is made up of four classes: healthy tissue, blood vessels, tumor tissue, and a background class designating the absence of any of the three classes mentioned above. To build up this map, three independent probabilistic maps are combined: the two outputs of the *HSI ResNet*, which are the brain cortex probability mask ($P_{ctx}$) and the blood vessel probability mask ($P_{vsl}$); and the probabilities provided by the given HSI tissue segmentator ($P_{clf}$).

One of the main purposes of this work is to improve the output of any HSI segmentation network capable of classifying with reasonable confidence at least healthy tissue samples while being aware that misclassifications may occur among the remaining classes. According to this principle, the probabilistic output of the HSI segmentator $P_{clf}$ is transformed into $Q_{clf}$ so that the probability associated with healthy tissue is maintained and the maximum among the remaining three probabilities is selected. The softmax function is then applied to the two resulting probabilities to sum to one. The transformation $Q_{clf}=T\left(P_{clf}\right)$ with $T:R^{4}\to R^{2}$ can be expressed as follows:

(3) $$Q_{clf}=\mathrm{softmax}\left(\left[P_{h},max\left\{P_{t},P_{v},P_{d}\right\}\right]\right)$$

where $P_{h},P_{t},P_{v}$, and $P_{d}$ are the corresponding probabilities of healthy, tumor, and vascular tissue and dura mater, respectively.

For the inpainting of the blood vessels in the final map, full priority is given to the blood vessel probability mask $P_{vsl}$ with respect to $Q_{clf}$. This means that in pixels where $P_{vsl}$ is greater than zero, the probabilities are distributed in such a way that $P_{vsl}$ always preserves its original value. On the other hand, the predicted cortex mask $P_{ctx}$ is used to filter the parenchymal area by setting zero any probability outside of its activation. The calculation of the final output map $P_{out}$ can, therefore, be expressed as

(4) $$P_{out}=\left[Q_{clf}\cdot \left(1-P_{vsl}\right)\cdot P_{ctx},P_{vsl}\cdot P_{ctx},1-P_{ctx}\right]$$

where the (·) operation represents an element-wise multiplication.

It is relevant to note that the first position inside the output map $P_{out}$ is still reserved for healthy tissue, the second for tumor, the third for blood vessels, and the fourth for background. In this case, visualization of the dura mater does not provide any information of interest for the final representation, so it is discarded.

## 4. Experiments and Results

To evaluate the proposed methodology, the two main aspects that comprise it are assessed: the impact the main stages of the multimodal training procedure have on the quality of the segmentation of the brain surface and its blood vessels in the HS dataset; and the effect on the classification metrics of the combination of cortical and tissue segmentation maps with respect to the base results provided by any HS segmentation network.

The structure of the common experimental setup begins with the definition of the experimental conditions (Section 4.1), the segmentation experiments conducted with the HS dataset, and the description of the comparative analysis with the methods selected from the literature (Section 4.3). The implementation details are provided in Section 4.5, and the metrics used to perform the quantitative and qualitative analysis are described in Section 4.7.

### 4.1. Experimental Setting

Following the standard procedure, the dataset described in Section 3.1.6 is randomly divided into test, validation, and training populations to perform 5-fold cross-validation. The test group consists of 13 out of the 67 available patients, representing 20% of them, and remains unchanged during cross-validation. Of the remaining 54 patients, 47 images form the training population, whereas 7 are dedicated to the validation (70% and 10% of the patient cohort, respectively). The training–validation division is performed randomly 5 times, obtaining the 5 different combinations, one for each fold. This distribution is kept for the complete set of tests conducted hereafter.

In order to establish a quantitative evaluation of the cortical and vascular segmentation in the HSI domain, the exposed brain surface of the 13 HS test captures is manually labeled. As a result, a gold standard reference is obtained on which the predicted cortex mask can be quantitatively evaluated. For the assessment of blood vessel segmentation, the GT originally annotated by the neurosurgeons is used without applying any densification or pre-processing.

In addition, the simplified brain surface annotation process described in Section 3.1.7, followed by the annotation refinement of Section 3.2.1, and the cortical perimeter approximation explained in Section 3.2.2 are performed in the HSI dataset. In this manner, a set of brain surface and vessels pseudo-label masks, analogous to the ones used in the RGB modality, are available to test an alternative fine-tuning process in the HSI domain. This set of pseudo-labels also allows us to establish comparative segmentation results between using the multimodal approach versus training a model using only the HSI cortical pseudo-labels.

### 4.2. Complementary Dataset: HELICoiD Database

To extend the validation of the proposed pipeline, the set of in vivo captures present in the HELICoiD database [9] is incorporated into the set of experiments. The HELICoiD database comprises 70 HS captures of 40 different patients also suffering from tumor-related conditions. The HS images were taken in different moments of the surgical intervention, including various stages of tumor resection. In this case, the HS camera used is a Headwall VNIR a-Series pushbroom scanner capable of capturing 826 bands contained in the spectral range between 400 and 1000 nm, with a maximum spatial resolution of 704×1004 pixels. After removing redundant spectral information and poorly illuminated regions of the spectrum, the HS cubes contain 128 bands in the range between 440 and 909 nm. The resulting cubes are denoised, applying a 5×5 mean filter and calibrated using a white reference to convert the sensor units into reflectance.

The set of 70 HS captures is also divided into training, validation, and test subsets for which 70%, 10%, and 20% of images are, respectively, assigned. The same 5 random partitions of training and validation images performed for SLIMBRAIN are conducted with the HELICoiD dataset, for which 54 and 7 images are dedicated for the training and validation sets, respectively. The remaining 14 images constitute a fixed test group for which the exposed brain surface is manually labeled to form the gold standard annotations. The tissue annotations performed by the neurosurgeons are also in a sparse format in a similar manner to the SLIMBRAIN ground truth. The summary of the number of labeled pixels for both databases can be seen in Table 2.

### 4.3. Comparison with Other Methods

The methodological frame followed in this letter is primarily based on the taxonomy developed in [4]. According to it, the method proposed in this work leverages the scarce and weak annotations that comprise the RGB and HSI datasets, combining the generation of reliable pseudo-labels with the masked loss functions exposed in Section 3.4, transferring the features learned in the RBG domain to the HS. To this extent, two main alternatives fitting the casuistry of the problem can be found in the literature:

- Structural and shape regularization helps compensate for the lack of complete annotations, guiding the model towards more coherent representations during training. In particular, the equivariance (EV) constraint approach [52] is commonly used to facilitate the model learning by ensuring the consistency between predictions of the transformed versions of the same image. In particular, the weakly supervised tumor segmentation methodology in PET/CT images proposed by G. Patel and J. Dolz [24] is adjusted to test the performance of the equivariance property as a regularization term in the training of the *HSI ResNet*. To do so, the BCE loss described in Section 3.4.3 is complemented with the mean squared error (MSE) loss between the prediction of the transformed images and the transformed prediction of the original set. The collection of applied transformations is made up of random flips and rotations.
- The second strategy aims to enforce coherence among embeddings corresponding to the same class prior to the generation of the segmentation mask. Therefore, the cosine similarity (CS)-based regularization approach developed by Huang et al. [23] for lymphoma segmentation in weak-annotated PET/CT images is also adopted as a complement to the base BCE loss explained in Section 3.4.3. Of special interest for this work is the self-supervised term of the loss function proposed in [23], which enforces the extracted features of the predicted tumor samples to be similar to each other but dissimilar to the non-tumor samples in terms of cosine similarity. This mechanism is adapted so that it can be applied to discriminate brain cortical pixels from the rest. The same idea is transferred to be used with the adjusted GT, so the base BCE loss function includes the self-supervised element just described and a weakly supervised regularization term.

In addition, among the approaches outlined in Section 2.6 that combine multiple image domains, four of them are of special interest to establish brain vessels and surface segmentation baselines: (1) MultiResUNet poses an alternative to the two-step method proposed in this work by combining in a single training both source and target domains. (2) CS-CADA also integrates in a single training stage the extraction of features from the source domain and the adjustment in the target modality. The main difference is the inclusion of domain-specific batch normalization and a contrastive learning strategy to ensure consistency between common elements. (3) MedSAM disposes of a model pre-trained with a vast collection of medical images, which gives it the ability to provide universal medical segmentation, as stated by the authors. (4) UniverSeg also features a fundation model capable of adapting to the target domain without a fine-tuning stage, requiring only a support set of labeled images.

As the four selected methods are designed to process three-channel images, the adaptation to the HSI domain is conducted using the RGB reconstructed version of the HS images. Also, as MultiResUNet, CS-CADA, and UniverSeg require fully labeled examples, the simplified brain surface annotations obtained for the HSI dataset along with the vessel pseudo-labels are used.

The comparative analysis between vessel pseudo-label extraction methods considers Vessel-CAPCTHA and Frangi filtering. For Vessel-CAPCTHA, both RGB and HSI datasets are labeled using its weak annotation process, obtaining the corresponding vessel pseudo-label masks for each dataset. To achieve a fair comparison with Frangi filtering, its *gamma* parameter is optimized using the Optuna framework, following the same procedure as in Section 3.2.3.

As the main purpose of the methodology proposed in this work is to increase the multiclass segmentation performance of brain tissue and tumor detection capability to any HS tissue segmentator, it is not intended to propose any novelty in this matter. Instead, two neural-network-based sample-wise classifiers that also deal with sparse annotations are selected from the benchmark conducted by Leon et al. [6], which explores the efficacy of different algorithms adapted for brain tumor detection using HS images:

- One-dimensional deep neural network (1D_DNN), proposed by Fabelo et al. [14] and designed to work at the HS single-pixel level through a two hidden layers structure with 28 and 40 neurons, respectively, and a final output layer, providing 4 different probabilities associated with each of the 4 tissues by softmax activation.
- Two-dimensional convolutional neural network (2D_CNN), presented by Hao et al. [12], which implements a ResNet-18 architecture for processing 11×11 overlapping patches extracted from the HS cube to obtain the probabilities belonging to the 4 tissues to be segmented also using softmax activation.

### 4.4. Evaluation Metrics

The performance of the model segmenting the cortical surface is evaluated in two aspects: The overlap between the predicted cortex mask and the gold standard, which is estimated using the DSC; and the distance between the boundaries of the cortex mask and the gold standard, assessed using the average symmetric surface distance (ASSD) [53].

The evaluation of cortical vessel segmentation is addressed in the same way as in Section 3.2.3 for the parameterization of the vascular pseudo-label generation procedure. The unmodified GT is used to calculate the vessel hit rate (VHR) between the set of pixels annotated as blood vessels $A_{V}$ and the predicted vessel mask $M_{V}$ in the following way:

(5) $$\mathrm{VHR}=\frac{1}{|A_{V}|}\sum_{h\in H}h\;\mathrm{where}\;H=M_{V}\cap A_{V}$$

where the VHR indicates the percentage of pixels labeled as blood vessels that have been correctly segmented, establishing its true positive rate (TPR) or sensitivity.

Similarly, the vessel error rate (VER) can be calculated between the pixels annotated as healthy or tumor tissue $A_{H,T}$ and the predicted vessel mask $M_{V}$ as follows:

(6) $$\mathrm{VER}=\frac{1}{|M_{V}|}\sum_{e\in E}e\;\mathrm{where}\;E=M_{V}\cap A_{H,T}$$

expressing the percentage of pixels belonging to the predicted vessel mask $M_{V}$ that incorrectly include actual healthy and tumor tissue samples, defining in this way its false positive rate (FPR).

The segmentation maps provided by the HS tissue segmentator and its refined versions are analyzed and compared using three different metrics commonly found in the literature: the area under the curve (AUC) of the receiver operating characteristics (ROC) [54], which allows comparisons of the classified samples in the presence of unbalanced datasets; the confusion matrix, to determine which classes are more prone to be mistaken for each other; and the F1 score:

(7) $$F1=\frac{2\times \mathrm{TP}}{2\times \mathrm{TP}+\mathrm{FP}+\mathrm{FN}}$$

where *TP*, *FP*, *TN*, and *FN* stand for true positives, false positives, true negatives, and false negatives, respectively.

Each of these three metrics is calculated for each class present in the GT for each patient. Once computed, global metrics (mAUC and mF1) can be determined by averaging the metrics per class for each patient and then obtaining the overall mean between the 13 test patients.

### 4.5. Implementation Details

To perform both cortical and tissue segmentation experiments in the HS domain, each HS pixel *x* of the dataset is normalized using a *min–max* scaling. Normalized HS pixels $\bar{x}$ are calculated as follows:

(8) $$\bar{x}=\frac{x-x_{min}}{x_{max}-x_{min}}$$

by extracting the minimum and maximum values $x_{min}$ and $x_{max}$ for each band of the validation and train sets, where $x_{min},x_{max}\in R^{25}$.

#### 4.5.1. Brain Cortex and Vessels Segmentation Training Details

The encoder pre-training described in Section 3.4.1 is performed for 400 epochs, applying a data augmentation strategy based on random compositions of equally probable transformations integrated by horizontal and vertical flips, color jittering and Gaussian blurring, each one of them applied with a probability of 50%. The batch size is set at 128, distributing its composition so that 1/3 are occupied by positive samples and the remaining 2/3 by negative samples. Inside the contrastive loss function, the temperature parameter τ is fixed at 0.1, as suggested in [46].

For the RGB encoder–decoder, the model is trained for 1000 epochs, but at every 10 epochs the DSC between the predictions of the brain surface and cortical vessels and their corresponding pseudo-labels is calculated in the validation set, saving the model that achieves the highest DSC on average between the vascular and surface segmentations. A batch size of 8 is chosen and a data augmentation procedure based on random compositions is also used. For this phase, the set of transformations is composed of horizontal and vertical flips, color jittering, Gaussian blurring, and random resized cropping. The random cropped region can cover an area within the proportion of [0.2,1.0] and an aspect ratio of [0.55,1.3] with respect to the original size of 217×409, to which is then rescaled.

In the HS fine-tuning stage, the same model selection strategy is used. In this case, the number of epochs is set to 700 and the average validation metrics are calculated with respect to the adjusted GT and vessel pseudo-labels explained in Section 3.4.3. Particularly, for the brain surface segmentation, the ACC between the predicted mask and the adjusted GT is computed, whereas the vessel mask is evaluated with respect to the masked vessel pseudo-labels using the DSC. Batch size is set to 8 too, but at this stage the data augmentation transformations are only made up with random horizontal and vertical flips.

For the three multimodal training steps, the AdamW [55] optimizer is used and cosine learning rate decay [56] is adopted starting at a value of 0.0001 and ending at 0.00001.

#### 4.5.2. HS Tissue Segmentation Network Training Details

All models used for the HS tissue segmentation task are trained using the AdamW optimizer, also making use of the cosine learning rate decay, starting at 0.001 and ending at 0.0001. A batch size of 8192 is employed and, given its magnitude, the LARS algorithm [57] is adopted. The model selection strategy is also based on the validation predictions performed each 10 epochs. In this case, the global ACC obtained for the four classes is the chosen metric.

#### 4.5.3. Software and Hardware Used

All experiments are performed on an A100 GPU with 80 GB VRAM (Nvidia Corporation, Santa Clara, CA, USA) using the Pytorch 2.4 library [58] to implement the described neural networks and the training procedure for both cortical and tissue segmentation tasks.

### 4.6. Quantitative Results

#### 4.6.1. Comparative Analysis of Neural Network Architectures for Cortical Segmentation

To evaluate the suitability of the proposed *HSI-ResNet*, its performance is contrasted with two popular autoencoder architectures: ResUnet++ [59] and MedNeXt [60]. The ResUNet++ model can be seen as the updated version of the ResUNet, therefore being a straightforward comparison with the custom adaptation proposed in this work. On the other hand, the MedNeXt architecture is selected for being the top performer in the medical image segmentation benchmark conducted in [61].

The results shown in Table 3 report the HSI segmentation metrics obtained by replicating the multimodal training methodology depicted in Figure 7 for each comparative NN architecture. Their encoder–decoder structure is used as backbone adding the projection head and the HSI stem for the pre-training and fine-tuning stages, respectively.

#### 4.6.2. Brain Surface and Cortical Vessels Segmentation

The quantitative analysis of the performance obtained on the brain surface and cortical vessel segmentation task using the proposed multimodal methodology and its comparison with the methods chosen from the state-of-the-art is summarized in Table 4. There, part (a) lists the combinations tested of the three training steps represented in Figure 7; part (b) reports how different variations of the base method compare to the methodology developed in this paper, part (c) shows the results obtained applying the comparative methods described in Section 4.3 to the HS dataset; and part (d) indicates the combination of the multimodal training procedure with the constraints of equivariance and cosine similarity, respectively. The DSC, ASSD, VHR, and VER displayed are calculated by averaging all individual values obtained for the five folds executed, for each one of the 13 patients present in the test group. The only exception applies to the first row, which corresponds to the metrics obtained using the vessel pseudo-label extractor detailed in Section 3.2 and parametrized according to Table S1. The values display the results from averaging the VHR and VER sourced from the estimated vascular masks for each patient in the test group. As no brain surface pseudo-label was extracted in the HS domain, it cannot be shown for any metrics in this regard.

The ablation study stated in part (a) of Table 4 is intended to compare the impact of the different stages of the training procedure, but also reveals differences between the use of HS or pRGB images when performing the cortical segmentations. The simpler training is conducted under the name of *HSI solo training*, where the *HSI ResNet* is trained only with the HS dataset using just the BCE loss function. To analyze the effects the encoder pre-training and the RGB pseudo-label-based autoencoder training stages have on the adjustment of the model separately, *Encoder pre-training + HSI fine-tuning* and *RGB training + HSI fine-tuning* experiments are conducted. On the other hand, the transferability to the HS domain of the two RGB training stages applied together is tested on *Encoder pre-training + RGB training*, where the results are extracted with the pRGB set of images without performing any fine-tuning with them or using the *HS stem*.

Part (b) of the table illustrates alternative design choices that can be embedded in the deployed method. The performance that can be achieved using only the HSI dataset with the refined brain surface annotations and vessel pseudo-labels is analyzed under the *HSI solo training with refined annotations* experiment. In addition, how these simplified annotations compare to the densified GT in the HS fine-tuning step is determined in the *Fully pre-trained with HSI refined annotations* experiment. The influence of performing the fine-tuning by feeding the pRGB images straight to the *RGB stem* is studied through the *Fully pre-trained + pRGB fine-tuning* experiment. The two remaining experiments of this part, *Frangi* and *Vessel-CAPTCHA*, explore the impact of replacing the vessel pseudo-labels obtained through the linear operator-based technique with these two approaches.

The ablation study first reveals the performance limitations caused by the scarcity of annotated samples. The *HSI solo training* experiment shows a pronounced drop in brain surface segmentation performance compared to the other training combinations analyzed. In contrast, the *Encoder pre-training + RGB training* comparison provides insight into the generalizability afforded by RGB pseudo-labels when transferred to the HSI domain.

The comparative performance between *Encoder pre-training + HSI fine-tuning* and *RGB training + HSI fine-tuning* indicates that the global information captured by the RGB pseudo-labels and exploited in the *RGB training + HSI fine-tuning* experiment has a greater impact on segmentation performance than encoder pre-training alone. However, the comparison between *RGB training + HSI fine-tuning* and *Fully pre-trained segmentation (proposed)* demonstrates that patch-based encoder pre-training establishes a stronger foundation for discriminating cortical from non-cortical tissue during subsequent training and refinement stages than omitting this pre-training step.

It is worth noting that the combination of different training stages primarily affects brain surface delineation performance. In contrast, blood vessel segmentation shows a significant improvement only when all training stages are jointly applied. Interestingly, the VER metric reaches its minimum in the *HSI solo training* and increases slightly by adding training stages in the RGB domain. This might find an explanation in some mislabeling introduced by the RGB vessel pseudo-labels, but also in the difference in sharpness between RGB and HS images. Sharper contours in the RGB domain may adjust the network to a higher sensibility, which translates into overdetections when applied to the HS images.

Regarding part (b) of Table 4, *Frangi* and *Vessel-CAPTCHA* outperform the vessel segmentation achieved by the proposed method at the cost of obtaining a higher VER. However, the results obtained in *HSI solo training with refined annotations* and *Fully pre-trained with HSI refined annotations* demonstrate the benefit of fine-tuning in the HSI domain using the densified GT.

The application of EV and CS constraints results in better metrics than *HSI solo training*, but both methods are outperformed when the HS fine-tuning is supported by a prior RGB fitting. Regarding details, HSI fine-tuning produces, by not a very large margin, better results than pRGB in DSC and VHR by increasing their mean values but also reducing the variability associated with the standard deviation, making it a more desirable option.

Part (d) of Table 4 evidences the robustness provided by the EV constraint when combined with the proposed methodology, achieving the highest DSC and ASSD and also the second highest VHR values with the lowest standard deviation for DSC. When contrasting the metrics obtained in the experiments *Proposed + CS* and the *Fully pre-trained segmentation (proposed)*, the lower DSC and ASSD of *Proposed + CS* can be explained by the lower weight given by the CS constraint to the spatial consistency of the segmentation and the greater importance placed on the closeness of embeddings. Nonetheless, this increased emphasis on similarity of the embeddings appears to contribute slightly to improving vessel detection.

#### 4.6.3. HS Tissue Segmentation

One of the main objectives of this work, the bettering of the segmentation provided by any HS tissue segmentator, is validated through the results shown in Table 5. In it, the sample-wise classification metrics obtained by each of the two neural networks tested and the resulting fusion between their tissue segmentation maps and the cortical segmentation provided by the *Proposed + EV* method (*1D DNN-F* and *2D CNN-F*), are compared in the *1D Difference* and *2D Difference* rows. The F1 and AUC scores are computed per class and globally for each one of the 13 patients in the test group, for the five folds performed.

**Figure 8:**
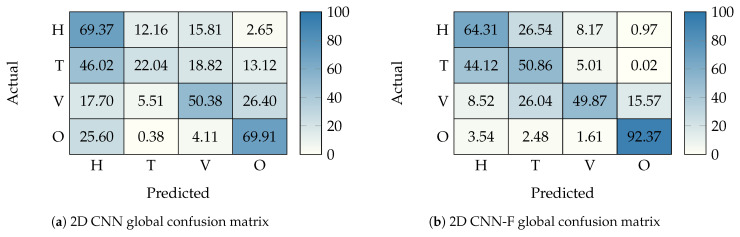
Comparison between the row-normalized confusion matrices corresponding to the greatest improvement by combining cortical and tissue segmentation probabilities. Both confusion matrices are expressed in percentage and are calculated by adding all annotated pixels in the five folds from the 2D CNN sample-wise classification results (**a**) and its fusion with the cortical segmentation obtained with the proposed methodology combined with the EV constraint (**b**). The H, T, V and O labels refer to healthy, tumor, and outside cortical area pixels. In the 2D CNN confusion matrix, the dura mater samples are taken as outside pixels.

Both *1D Difference* and *2D Difference* rows expose a systematic improvement in mean F1 and AUC for all classes and in their global calculation. However, the high standard deviation present in all the means calculated reveals that in some cases, a worsening of the results may occur, especially if the HS tissue segmentator mistakes a large number of healthy pixels for any other class. This fact can be better understood by looking at the row-normalized confusion matrices shown in Figure 8. It can be seen that the majority of misclassified healthy and vessel pixels in matrix Figure 8a are concentrated as tumor false positives in matrix Figure 8b. However, it should be noted that the percentage of correctly classified tumor pixels doubles when applying the probability fusion process, while the detection of pixels outside the cortical area improves by 22%.

When addressing the discrepancy between the percentage of correctly predicted vascular samples in Figure 8b and the VHR reported in Table 4, the reason can be found in the prevalence of a certain number of FN vessel samples in its segmentation mask. Whereas column *V* in Figure 8b shows a significant reduction in vessel FP pixels compared to the same column in Figure 8a, relative percentage of vessel FN remains practically the same. This aspect translates into a high vessel TP rate while keeping the vessel FN barely unaltered. Given the definition of the F1 score expressed in Equation (7), the gain in TP rate while keeping a similar value of FN causes an increase in the F1 score that is not as high as the VHR values from Table 4 could suggest.

#### 4.6.4. Brain Surface and Cortical Vessels Segmentation on the HELICoiD Database

The characteristics of the HELICoiD dataset impose certain limitations on the application of the proposed methodology. As the chosen NN can only process images with size 217×409, the main adaptation that must be performed is to adjust the spatial resolution of the HS cubes so that the *HSI ResNet* can process them. To this end, the HS cubes are rotated so that the shorter edge is in a vertical position and, if the shorter edge is still larger than 217 pixels, the cube is resized accordingly. The rest of the HS image is padded with zeros, resulting in an HS cube with size 217×409 and 128 bands.

The set of vascular and cortical pseudo-labels can be obtained in the same manner as described in Section 3.2. However, as the HELICoiD database does not have a complementary set of RGB images, pseudo-label generation is limited to the HS images only. Therefore, the set of cortical segmentation experiments that can be conducted is summarized in Table 6. For both experiments, the encoder and the decoder were pre-trained with the SLIMBRAIN RGB dataset. For the *HSI solo training with refined annotations* experiment, the manual annotation process described in Section 3.1.7 is conducted, producing the set of brain surface pseudo-label masks needed for this training variant. To obtain the final metrics reported in Table 6, the 217×409 output masks undergo an inverse resize and cropping process to restore the original image dimensions. This allows the brain surface and vascular metrics to be computed using the annotations defined on the original-sized HS cubes.

A comparison between Table 4 and Table 6 reveals a noticeable drop in brain surface segmentation performance. As discussed later, Section 4.7 shows that the HELICoiD images are entirely focused on the craniotomy site, resulting in a reduced spatial context of the scene. The absence of surrounding guiding elements may negatively affect brain surface segmentation. In contrast, blood vessel segmentation performance remains comparable to that obtained with the SLIMBRAIN dataset.

#### 4.6.5. Tissue Segmentation on the HELICoiD Database

The analysis of tissue segmentation refinement for the HELICoiD dataset is presented in Table 5. The vascular and cortical masks used to refine the tissue segmentation maps were obtained following the *Proposed + EV* training procedure, as it achieved the best performance among the two methods evaluated in Table 6. The same models used in Section 4.6.3 are now trained on the HELICoiD cubes, with no size adaptation required. This is because the 1D model processes batches of HS pixels, while the 2D model only requires 11×11 patches; consequently, both NN architectures are independent of the global image size.

As the tissue segmentation maps maintain the original spatial size of the HS cubes, the probability fusion step is performed after applying the inverse resizing and cropping process described in Section 4.6.4. The resulting metrics of the refined tissue segmentation maps can be seen in rows *1D DNN-F* and *2D CNN-F* from Table 7.

One notable observation is the discrepancy between the F1 score and the AUC metric for the 1D DNN model. Probability fusion achieves a mean improvement of 29.38 in the F1 score for the tumor class, but this gain translates into a 9.05 decrease in AUC for the same class. The root of this matter may be found in the severe imbalance of the number of labeled tests tumor samples against the rest of the classes (see Table 2). The low tumor F1 score in 1D DNN may indicate a high number of false negative predictions for the tumor class, while still providing relatively informative probability estimates, which can lead to higher AUC values.

The experiments with the 2D CNN pose a scenario in which the proposed methodology loses its effectiveness. The higher vascular detection capability of the 2D CNN, combined with a more robust tumor segmentation performance, makes the contribution of the cortical segmentation masks counterproductive for improving the tissue segmentation map. In this situation, the 128 spectral bands present in the HELICoiD HS cubes may enable the tissue segmentation network to outperform the cortical segmentation network.

#### 4.6.6. Computational Performance

The feasibility of a clinical implementation of the proposed pipeline is also analyzed in terms of computational requirements. To this end, the time required to segment the brain surface and its blood vessels, how long it takes to complete tissue segmentation with the two NN evaluated, and the time spent in the probability fusion stage are measured and presented in Table 8. In order to set a realistic scenario, a machine with standard specifications is used. In this case, the computer has an Intel i5-10400F processor, 32 GB of RAM, and an NVIDIA GTX 1650 with 4 GB of VRAM. The code used in this benchmark was written in Python without particular optimizations.

The values reported in Table 8 were obtained by averaging the time measured for each individual image in the test set. In the case of the tissue segmentation measurements, the batch size number indicates that the HS cube could not be processed at once, so it was split in batches of a maximum size of 2048 elements.

### 4.7. Qualitative Results

**Figure 9:**
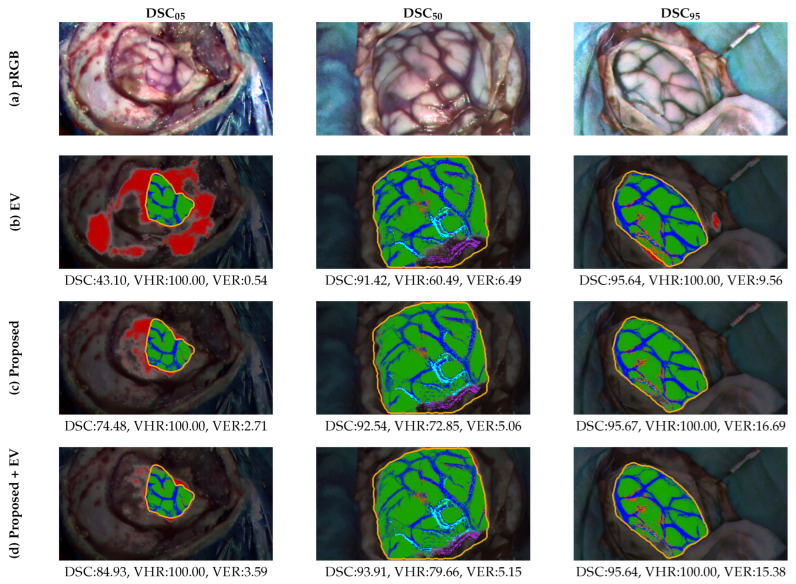
Comparison between pseudo-RGB images (**a**) and different segmentation maps obtained with the equivariance constraint approach alone (**b**), the proposed multimodal training methodology using the three training steps described in Section 3.4 (**c**), and the combination of the proposed methodology with the equivariance constraint (**d**). Images are selected according to the 5th, 50th, and 95th quantile averaging the DSC obtained by the three models for each image. Inside each segmentation map are **yellow**: gold standard, **green**: correctly segmented cortical area, **red**: region wrongly included as brain cortex, **blue**: vessel segmented mask, **light blue**: blood vessel pixels from the GT included in the segmented vessel mask (TP), **orange**: healthy or tumor pixels from the GT wrongly included in the segmented vessel mask (FP), **purple**: blood vessel pixels from the GT not included in the segmented vessel mask (FN).

**Figure 10:**
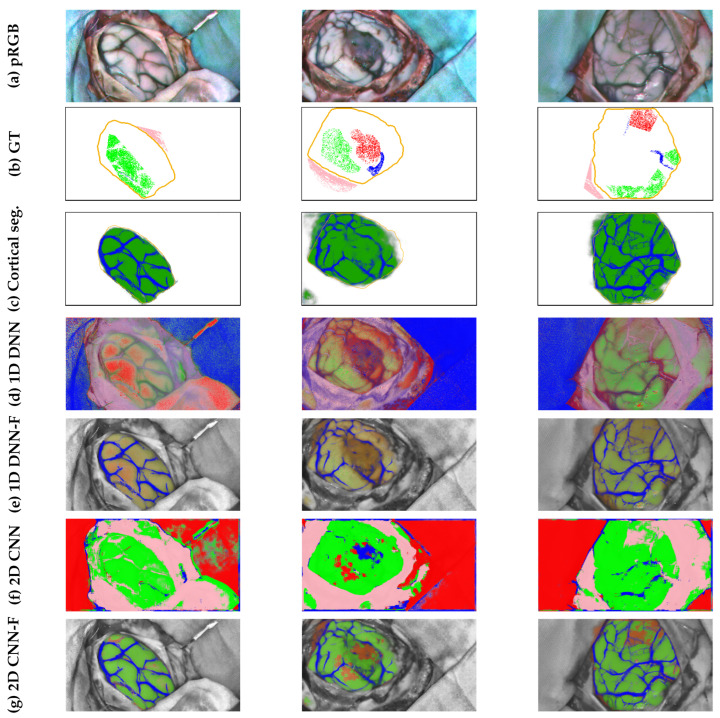
Examples of the tissue segmentation results obtained on the SLIMBRAIN database using the two neural networks described in Section 4.3, showing (**a**) the pseudo-RGB images of the segmented HS cubes, (**b**) the corresponding GT including the gold standard marked in yellow, (**c**) the result of the brain surface (green) and vessel (blue) segmentation with the gold standard reference (yellow) obtained using the proposed methodology combined with the EV constraint, (**d**) the probabilistic tissue segmentation map produced by the 1D DNN, (**e**) the combination between the cortical segmentation and the 1D DNN tissue segmentation map, (**f**) the probabilistic tissue segmentation map produced by the 2D CNN, and (**g**) the combination between the cortical segmentation and the 2D CNN tissue segmentation map. Green, red, blue, pink and gray colors in rows (**d**–**g**) refer to healthy tissue, tumor tissue, blood vessels, dura mater, and non-brain surface region, respectively.

To complement the numerical analysis performed in Section 4.6.2 and Section 4.6.3, Figure 9 and Figure 10 provide visual information on the quality and appearance of the cortical and tissue segmentation results.

Starting with the brain surface and vessel segmentation task, examples of combined cortical and vascular maps are shown in Figure 9. As the cortex gold standard is the most complete annotation available in the HS dataset, the qualitative organization of the results is based on the cortex DSC metric using the ranking procedure proposed by Seidlitz et al. [5], which is applied to select illustrative examples to establish a comparison between different models that predict the same test set. In the case of this work, the models being compared are those corresponding to the experiments *Fully pre-trained segmentation (proposed)*, *Equivariance*, and *Proposed + EV* from Table 4 with the DSC obtained in the cortical segmentation of each patient ranked in three categories: bad, intermediate, and good. These categories refer to the 5th, 50th, and 95th percentiles estimated from the average DSC of the three models for each image in each fold. This can be interpreted as meaning that a certain image of a given fold placed in the 5th percentile is, on average for the three models, better than 5% of the averaged predictions and so on for the 50th and 95th percentiles.

For the HS tissue segmentation task, Figure 10 displays three examples of patients with different brain tumor conditions, two of them with emerging tumors (central and right column patients). In this figure, the improvement in the tissue segmentation maps for the proposed methodology is shown. Especially in row Figure 10g, the cortical and vascular segmentations performed by the *Proposed + EV* model demonstrate its capability to correct blood vessel pixels misclassified as healthy and dura mater, and to rectify the tumor samples mistaken for vascular tissue and dura mater. This example condenses the idea and motivation of the present work, which is that, as long as the HSI tissue segmentator is capable of an adequate healthy tissue identification, the proposed methodology can significantly improve the tumor detection. Another aspect worth mentioning is that, through the probability fusion between tissue and cortical segmentation maps, a more readable representation is achieved. This can be observed when comparing rows Figure 10d,f with their corresponding fused results in rows Figure 10e,g, where rows Figure 10e,g allow easier interpretation of the tissue segmentation of the craniotomy scene.

**Figure 11:**
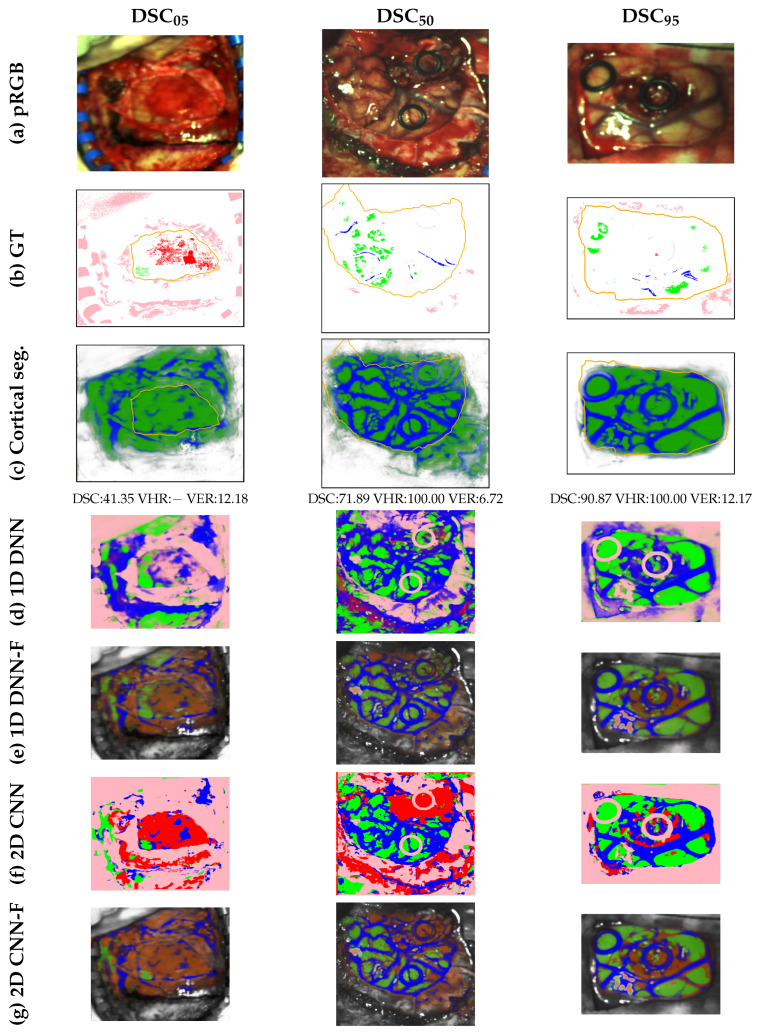
Tissue segmentation results on the HELICoiD database using the two neural networks described in Section 4.3, showing (**a**) the pseudo-RGB images of the segmented HS cubes, (**b**) the corresponding GT including the gold standard marked in yellow, (**c**) the result of the brain surface (green) and vessel (blue) segmentation with the gold standard reference (yellow) obtained using the proposed methodology combined with the EV constraint, the DSC, VHR and VER obtained in these images, (**d**) the probabilistic tissue segmentation map produced by the 1D DNN, (**e**) the combination between the cortical segmentation and the 1D DNN tissue segmentation map, (**f**) the probabilistic tissue segmentation map produced by the 2D CNN, and (**g**) the combination between the cortical segmentation and the 2D CNN tissue segmentation map. Green, red, blue, pink and gray colors in rows (**d**–**g**) refer to healthy tissue, tumor tissue, blood vessels, dura mater, and non-brain surface region, respectively.

Similarly to Figure 10, the graphical information for the results obtained on the HELICoiD database is shown in Figure 11. This figure presents examples organized according to their brain surface DSC percentile. The particular DSC, VHR and VER values for the images displayed are reported below the cortical segmentation examples in row Figure 11c.

## 5. Discussion

The work presented in this paper addresses the challenge of improving multiclass segmentation of brain tissue using HS imaging with the ultimate goal of detecting regions affected by a brain tumor. To this end, a methodology is established to segment the brain surface and its cortical vessels by applying a multimodal transfer learning approach from RGB to HS, based on weak annotations of both domains.

**Figure 12:**
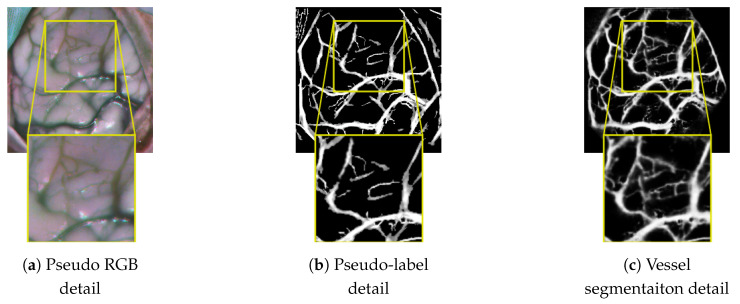
Comparison between the fine vessel detail of a pseudo-RGB image (**a**), the pseudo-label generated for those vessels (**b**) and the vessel test segmentation of that region using the *Proposed + EV* model (**c**).

Reasonable doubts may arise when deciding whether to use the vessel pseudo-label generation method proposed in Section 3.2.3 or the vessel segmentation map provided by the *HSI ResNet*. Although the vascular pseudo-label generation offers a robust mechanism for capturing the majority of the blood vessels, it does not always cover the complete range of thin vessels. However, the capillaries it is able to detect leave sufficient clues for the network to learn to capture these contours without incurring overdetections. This matter is represented in Table 4, where the VHR obtained using the *Vessel pseudo-labels* method is outperformed by any other listed method. In particular, each vessel segmentation method trained with pseudo-labels improves, on average, by 13.11±1.46 on the VHR obtained by the *Vessel pseudo-labels* on the test set, while it worsens, on average, by 7.34±0.70 on the VER. Achieving almost twice as much improvement in VHR as deterioration in VER represents a positive trade-off in favor of the proposed training method based on pseudo-labels. Figure 12 illustrates a comparison between the details of a segmentation mask predicted by the *HSI ResNet* from the test population and the pseudo-label generated in the same region, showing better sensitivity to thin vessels in the segmentation mask.

With regard to the multimodal aspect of the proposed training methodology, the results obtained highlight the usefulness of combining an NIR HS camera with a high-resolution RGB camera in the same acquisition system. The transferability between the detailed information captured by the RGB camera into the HS domain proved its worth, specifically when analyzing the cortex and vessel segmentation metrics that Table 4 illustrates. Inside part *(a)*, it can be appreciated that, while all methods show close behavior segmenting vascular tissue, the brain surface scores obtained using the *HSI solo training* are greatly outperformed by all methods that combine both RGB and HS image sources. It is also worth mentioning the fact that the models trained with a more complete supervision, such as that provided by the simplified annotations in the HSI domain (*HSI solo training with refined annotations* in Table 4), perform substantially worse in terms of accurate brain surface and vessel segmentation when no RGB source of information is used. Additionally, the proposed multimodal methodology can be compared to the work elaborated by Fabelo et al. [14], where a similar approach was applied to improve tissue segmentation maps for brain tumor detection. In that work, a segmentation of the brain surface and cortical vessels was also performed, but, in this case, by using only a fully annotated brain cortex HS dataset produced by a visible and near-infrared (VNIR) pushbroom camera. The brain cortex DSC reported in [14] was 86.5, compared to the 92.08 achieved by the best-performing model of the present work.

Translating this analysis into the HELICoiD database, the model seems to struggle to properly segment the brain surface, dropping the average DSC to 77.59 in the best performing scenario. The increase in ASSD to 32.47, combined with a high standard deviation of 24.33, is particularly insightful, as it suggests the presence of images in which the boundaries of the segmented cortical masks extend well beyond the true anatomical limits. The first and second columns of Figure 11 illustrate the issue described. As the HELICoiD database incorporates a wider variety of surgical scenarios, the presence of blood over the surrounding dura mater can be misleading for the cortical segmentation network. Therefore, this effect becomes increasingly pronounced as tumor resection progresses.However, the third column of Figure 11 demonstrates that the proposed method can achieve adequate segmentation under conditions that differ from those studied in the SLIMBRAIN database.

Focusing on the HS tissue segmentation task on the SLIMBRAIN database, it can be observed in Table 5 that the comparison between the original and the fused results shows a significant increase in its healthy AUC score but shows a rather negligible improvement in healthy F1 score for both NNs evaluated. This may suggest that the total quantity of healthy samples correctly identified remains practically unaltered by the probability fusion, whereas the increase in healthy AUC could be an indicator of more confidence placed on these predictions. In either case, healthy tissue identification is an aspect that is not intended to be directly improved by the proposed methodology. However, it is noteworthy how the increase in tumor metrics is not constant across the predictions of the two tissue segmentators tested. Particularly, while the application of the correcting strategy improves the *1D DNN* tumor F1 metric by 8.24 points, it induces an increase of 15.48 for the *2D CNN*. These observations may indicate that the maximum achievable improvement in tumor detection does not depend on the initial tumor prediction of the tissue segmentator, but, rather, on its performance in the healthy class. This conjecture is supported by the fact that even though average tumor F1 scores for *1D DNN* and *2D CNN* differ by 10.92 points, its fused metric only diverges by 3.68 points. This could, therefore, be a confirmation of the underlying hypothesis of this work, namely, that the task of detecting cortical brain tumor tissue can be improved by simplifying the decision between healthy and unhealthy pixels once all other sources of error have been suppressed.

The complementary experiments performed on the HELICoiD database reveal a crucial point: when tissue segmentation outperforms cortical segmentation, the proposed pipeline becomes ineffective. In essence, improvements in the tissue segmentation network increase the level of precision required from the cortical segmentation network. This consequence is observed in experiments involving the 2D CNN neural network, where the tumor F1 score reaches a value of 51.10. In this situation, even though the VER obtained in cortical segmentation is contained at 5.22%, the small number of labeled tumor samples greatly penalizes any vessel segmentation mistake that overrides correctly classified tumor pixels.

The effective reduction in the demands placed on the tissue segmentation network suggests that its computational complexity could also be reduced. Therefore, an NN architecture specialized in distinguishing healthy from non-healthy tissue could be proposed, exploring in this specialization which bands are the most discriminative for this task in a manner similar to the study conducted by B. Martinez et al. in [63]. This further development could impact the computational times shown in Table 8, as the cortical segmentation network could likewise benefit from the insights gained through this band selection analysis.

### Limitations

The analysis conducted in Section 4.6 and Section 5 shows certain strengths of the procedure developed in this investigation, but also demonstrates certain limitations inherent in the chosen solution. The one that most affects the final segmentation result is related to obtaining pseudo-labels in both RGB and HS modalities. The absence of any kind of medical annotations in the RGB dataset makes the pseudo-label generation process subject to some risks related to the accuracy of the extracted labels and does not completely guarantee the applicability to other kinds of dataset. These risks manifest themselves in the form of inaccurate identification of the boundaries of the cerebral cortex and the inclusion of contours that do not belong to vascular tissue as mislabeling. Regarding vascular pseudo-labels, the qualitative results obtained on the HELICoiD database and presented in Figure 11 evidence these issues. In particular, Figure 11c in the first column shows how the model mistakes darker parts of the brain surface as vascular tissue. As the generation of the blood vessel pseudo-labels focuses purely on the morphology and brightness of the contours, it lacks discriminability when differentiating necrosed or damaged tissue from actual blood vessels. As a result, this kind of mistake affects the identification of the true tissue underlying. Conversely, if the tissue segmentation network overextends the segmented vascular regions, the excess pixels that are not identified as blood vessels by the cortical segmentation network may be reassigned as tumor samples. This effect would increase the number of tumor false positives, resulting in misleading tissue segmentation maps.

Regarding the performance drop experienced in the 2D CNN probability fusion using the HELICoiD dataset, it is reasonable to consider potential improvements for the *HSI ResNet*. While the cortical segmentation results might be satisfactory on the SLIMBRAIN dataset, it is clear that the proposed neural network architecture does not fully exploit the information provided by the 128 spectral bands of the HELICoiD captures. One possible improvement would be to modify the *HSI Stem* to incorporate 3D convolutional layers, which could more effectively leverage the spectral–spatial information.

Another aspect to be considered is that, given the sparsity and low number of vessel samples, the parameterization of the HS pseudo-label extraction suffers from certain uncertainty that may affect the optimal performance of the model. This uncertainty is also present in the analysis of the results, where incomplete blood vessel annotations require deeper inquiry to draw valid conclusions.

Finally, the fusion of cortical and tissue segmentation maps has been proven to be able to bring improvements to segmentation representation, but also, in the case of a flawed segmentation where healthy tissue is mistaken for blood vessels and dura mater, the combination with the segmentation map cannot remedy it. Moreover, the combination of probabilities will cause these defective samples to appear as tumor false positives. This is the main reason to consider exploring a binary HS tissue segmentator that is reliable enough to avoid these errors.

## 6. Conclusions

This study explored a relatively novel strategy aimed at improving brain tumor detection in HS images by simplifying the segmentation problem through a reduction in the complexity of elements to be segmented by any given HS tissue segmentator. The multimodal training procedure, backed by a simple yet effective pseduo-label generation process, produced models transferable from the RGB to the HS domain capable of providing robust segmentation of the brain surface and its blood vessels on the SLMIBRAIN database. The combination of cortical and tissue segmentation maps resulted in an improvement in tumor detection metrics in those cases where the initial tissue segmentation was insufficient. Further development and refinement of the proposed methodology could, therefore, enable accurate tumor segmentation while providing an easily interpretable representation map for medical diagnosis assistance.

The improvement in tumor segmentation observed in Table 5 also suggests the potential to develop different approaches that will be examined in the future. For example, one could explore the possibility of performing the cortical segmentation in the RGB domain, leveraging its higher spatial resolution. Then, the in-depth information captured by the acquisition system could be used to reproject the resulting masks into the HS image, applying, then, the proposed probability combination with the HS tissue segmentation.

The extraction of reliable blood vessels and brain surface masks could also contribute to a more efficient labeling process for specialists. This would simplify the task of labeling only the regions affected by the tumor, thereby producing a more dense and complete ground truth for tissue segmentation.

## Figures and Tables

**Table 1:** Number of pixels per class of the annotated HS images.

	Healthy	Tumor	Blood Vessels	Dura Mater	Total
Annotated pixels	182,314	32,702	18,662	80,312	313,990

**Table 2:** Number of pixels per class and population of the annotated HS images for SLIMBRAIN and HELICoiD databases.

Partition	SLIMBRAIN				HELICoiD			
	Healthy	Tumor	Vessel	Other	Healthy	Tumor	Vessel	Other
Val-train pix.	146,986	28,702	13,587	65,944	262,770	31,389	113,853	387,854
Test pix.	35,328	4000	5075	14,368	60,381	10,899	17,851	135,720
Total pix.	182,314	32,702	18,662	80,312	323,151	42,288	131,704	523,574

**Table 3:** Quantitative comparison of the proposed *HSI-ResNet* with ResUNet++ and MedNeXt for brain surface and vascular segmentation in the HSI domain.

NN Model	Cortex		Vessel	
	DSC	ASSD	VHR	VER
ResUNet++ [59]	78.14±18.84	16.59±15.03	94.39±12.21	8.29±4.60
MedNeXt [60]	89.91±10.48	8.60±8.87	95.29±8.25	9.93±5.78
HSI-ResNet	91.34±6.33	8.21±7.92	94.93±8.12	9.48±5.03

**Bold** values indicate the best result.

**Table 4:** Comparison of the brain surface and cortical vessel metrics obtained on the test set with the brain surface and vessel pseudo-labels generation methods proposed in Section 3.2.1 and Section 3.2, ablation study of the training steps described in Section 3.4 (a), different variations of the elements involved in the proposed methodology (b), results obtained with the six comparative methods (c), and the combination of the proposed methodology with the cosine similarity and equivariance approaches (d).

	Method	Cortex		Vessel	
		DSC	ASSD	VHR	VER
	Vessel pseudo-labels	-	-	80.57±19.00	1.63±1.43
	HSI refined annotations	93.24±3.55	4.37±1.89	-	-
(a)	HSI solo training	78.13±21.29	22.99±16.01	92.99±12.58	8.26±4.78
	Encoder pre-training + HSI fine-tuning	81.19±14.72	19.40±12.84	92.98±12.41	9.00±5.46
	Encoder pre-training + RGB training	84.88±10.45	9.74±6.00	91.03±12.56	9.07±4.99
	RGB training + HSI fine-tuning	88.46±11.53	11.59±14.41	92.82±13.45	9.20±5.02
(b)	HSI solo training with refined annotations	87.87±10.46	11.81±10.32	78.71±22.73	6.91±4.24
	Fully pre-trained with HSI refined annotations	89.06±7.93	9.62±9.43	80.34±21.79	6.40±3.79
	Fully pre-trained + pRGB fine-tuning	89.90±9.31	7.43±5.12	94.74±10.10	10.42±5.75
	Vessel-CAPTCHA [27]	90.41±7.61	9.47±11.28	97.59±8.10	13.18±7.25
	Frangi [18]	91.29±6.54	7.47±5.88	96.55±7.99	10.98±5.86
(c)	UniverSeg [32]	78.12±4.82	8.52±3.95	84.61±53.29	9.13±4.78
	MultiResUNet [62]	80.25±13.06	13.32±11.14	78.67±23.70	7.62±4.99
	Cosine similarity [23]	80.73±19.13	21.37±17.18	93.79±7.90	7.67±4.85
	CS-CADA [29]	80.78±7.11	15.06±5.41	62.25±52.49	6.55±5.73
	Equivariance [24]	82.13±18.97	18.38±16.08	92.19±13.18	8.48±4.73
	MedSAM [33]	88.21±5.69	11.41±6.70	75.95±52.42	16.31±9.47
	Fully pre-trained segmentation (proposed)	91.34±6.33	8.21±7.92	94.93±8.12	9.48±5.03
(d)	Proposed + CS	89.49±10.74	11.96±15.11	95.86±7.72	8.96±4.90
	Proposed + EV	92.08±5.87	7.91±10.18	95.42±8.28	9.19±4.86

**Bold** values indicate the best result.

**Table 5:** Comparison between the 1D DNN and the 2D CNN sample-wise classification performance in the test set. *1D DNN* and *2D CNN* rows indicate the scores obtained with the original tissue segmentation maps outputted by both NNs. *1D DNN-F* and *2D CNN-F* express the metrics achieved when fusing the original tissue segmentation maps with the cortical segmentation produced by the proposed methodology plus the EV constraint. *1D Difference* and *2D Difference* display the respective subtraction of the original rows from the fused for each NN. (**H** = healthy, **T** = tumor, **V** = vessels, **O** = outside area, **mF1** = mean F1 and **mAUC** = mean AUC).

Method	F1					AUC				
	H	T	V	O	mF1	H	T	V	O	mAUC
1D DNN	67.40	28.70	24.98	60.94	62.68	85.24	68.67	84.43	93.08	86.07
	±28.65	±22.73	±26.69	±29.54	±23.73	±20.44	±14.86	±13.93	±11.70	±14.34
1D DNN-F	67.96	36.94	38.98	89.78	72.69	91.98	76.08	91.00	98.69	93.08
	±29.74	±28.13	±20.84	±15.24	±23.11	±10.47	±15.63	±8.19	±2.82	±7.76
1D Difference	0.57	8.24	14.90	28.84	10.02	6.74	7.41	5.78	5.61	7.01
	±8.32	±10.55	±27.63	±33.65	±18.98	±13.78	±15.08	±12.39	±12.32	±9.88
2D CNN	68.24	17.78	28.47	74.55	66.84	78.66	58.57	77.41	91.31	81.17
	±28.49	±22.54	±19.05	±24.26	±24.03	±20.19	±25.14	±19.31	±15.59	±17.56
2D CNN-F	69.17	33.26	39.32	89.57	73.30	92.28	73.54	91.00	98.69	93.38
	±28.07	±26.28	±21.06	±15.67	±20.85	±8.68	±12.39	±8.19	±2.82	±6.26
2D Difference	0.93	15.48	11.53	15.02	6.46	13.62	14.97	12.8	7.39	12.21
	±6.71	±25.28	±19.15	±24.19	±13.51	±16.85	±19.68	±17.24	±15.91	±15.22

**Table 6:** Brain surface and blood vessels segmentation results obtained using the HELICoiD database.

Method	Cortex		Vessel	
	DSC	ASSD	VHR	VER
HSI solo training with refined annotations	75.66±19.55	37.73±28.63	94.72±8.37	6.49±6.21
Proposed + EV	77.59±16.94	32.47±24.33	99.56±1.60	5.22±3.30

**Bold** values indicate the best result.

**Table 7:** Comparison between the 1D DNN and the 2D CNN sample-wise classification performance in the HELICoiD test set, evaluating the same metrics as in Table 5. The cortical segmentation maps employed for the probability fusion are obtained through the *Proposed + EV* experiment in Table 6. (**H** = healthy, **T** = tumor, **V** = vessels, **O** = outside area, **mF1** = mean F1 and **mAUC** = mean AUC).

Method	F1					AUC				
	H	T	V	O	mF1	H	T	V	O	mAUC
1D DNN	86.88	2.52	89.37	94.00	90.19	99.82	94.33	98.91	96.48	96.84
	±21.17	±11.9	±12.4	±6.87	±8.82	±0.31	±5.26	±2.82	±8.11	±7.13
1D DNN-F	90.27	31.90	72.19	90.80	89.39	99.27	85.28	96.87	99.45	97.99
	±16.46	±22.0	±18.21	±11.65	±7.37	±1.08	±13.82	±3.97	±1.41	±3.5
1D Difference	3.39	29.38	−17.18	−3.20	−0.80	−0.55	−9.05	−2.04	2.97	1.15
	±2.58	±4.51	±2.45	±3.92	±2.16	±0.22	±3.15	±0.2	±0.43	±0.22
2D CNN	89.75	51.10	83.72	94.55	93.31	99.72	96.88	99.03	99.22	99.20
	±18.21	±32.85	±25.57	±9.54	±8.69	±0.54	±3.9	±4.63	±2.66	±2.18
2D CNN-F	91.00	38.65	72.06	90.41	89.53	99.36	87.92	96.87	99.45	98.14
	±13.63	±25.96	±18.67	±12.6	±7.47	±0.99	±12.64	±3.97	±1.41	±3.44
2D Difference	1.25	−12.45	−11.66	−4.14	−3.78	−0.35	−8.96	−2.15	0.22	−1.05
	±1.86	±8.32	±2.37	±4.92	±2.9	±0.24	±2.23	±1.33	±0.47	±0.3

**Table 8:** Computational times measured in different stages of the tissue segmentation refinement pipeline for both SLIMBRAIN and HELICoiD datasets.

Batch Size	Cortical Seg.	Tissue Seg. (1D DNN)	Tissue Seg. (2D CNN)	Prob. Fusion
	1 (Complete Cube)	2048 HS Pixels	2048 HS Patches	1 (Complete Cube)
Time (s) with SLIMBRAIN	0.011±0.008	0.092±0.321	37.091±0.378	0.020±0.003
Time (s) with HELICoiD	0.109±0.408	0.124±0.373	40.276±0.907	0.019±0.007

## Data Availability

All the in vivo hyperspectral human brain data used in this study is present in the SLIMBRAIN database, which is available at https://slimbrain.citsem.upm.es/ (accessed on 10 December 2025). Note that access must be granted, under reasonable request, before downloading the data. The source code can be found at: https://gitlab.citsem.upm.es/public-projects/hyperspectral/cortical_segmentation (accessed on 26 February 2026).

## Supplementary Materials

The following supporting information can be downloaded at: https://www.mdpi.com/article/10.3390/cancers18050857/s1, Section S1: RGB brain cortex annotation refining; Section S2: Cortical vessel pseudo-label generation; Section S3: HSI ground truth densification and background complementation; Algorithm S1: Expansion of manual annotations; Table S1: Optimized parameters using the Optuna framework to generate cortical vessel pseudo-labels with the HS dataset. Both kernel parameters indicate the size in pixels per size of the linear operators contained in the kernel. References [64,65,66,67,68] are cited in the Supplementary Materials.
