# Supplementary material for: Improving Brain Tumor Detection by Cortical Surface and Vessels Segmentation Through RGB-to-HSI Transfer Learning
Source: Cancers (Basel). 2026 Mar 6;18(5):857. doi: 10.3390/cancers18050857 (PMC12984245; doi:10.3390/cancers18050857)
Supplement: Supplementary file 1 [file cancers-18-00857-s001.zip › cancers-4138329-supplementary.pdf]

cancers

MDPI

Article

Improving Brain Tumor Detection by Cortical Surface and Vessels Segmentation Through RGB-to-HSI Transfer Learning

Guillermo Vazquez [1,*], Alberto Martín-Pérez [1], Angel Perez-Nuñez [2,3,4], Alfonso Lagares [2,3,4], Eduardo Juarez [1] and Cesar Sanz [1]

[1] Research Center on Software Technologies and Multimedia Systems, Universidad Politécnica de Madrid (UPM), 28031 Madrid, Spain; a.martinp@upm.es (A.M.-P.)
[2] Neurosurgery Department, Hospital Universitario 12 de Octubre, 28041 Madrid, Spain
[3] Medicine Faculty, Universidad Complutense de Madrid (UCM), 28040 Madrid, Spain
[4] Instituto de Investigación Sanitaria Hospital 12 de Octubre (Imas12), 28041 Madrid, Spain
* Correspondence: guillermo.vazquez.valle@upm.es

Keywords: hyperspectral; segmentation; transfer learning; vessel segmentation; cortical segmentation

S1. RGB brain cortex annotation refining

This section provides the implementation details for the refinement process of the simplified cortical annotations described in Section 3.2.1 of the original manuscript. For better characterization, Algorithm S1 includes the pseudocode that summarizes this process.

Algorithm S1: Expansion of manual annotations

Input: RGB image: $\mathcal{I}_{RGB}$, Manually annotated mask $\mathcal{M}$
Output: Refined cortical mask $\mathcal{M}_{ref}$
$\mathcal{I}'_{RGB} \leftarrow$ Crop $\mathcal{I}_{RGB}$ centered at $\mathcal{M}$
Edge image $\mathcal{E} \leftarrow$ Apply Cany algorithm to $\mathcal{I}'_{RGB}$
$\mathcal{I}_{YCbCr} \leftarrow$ Adjust contrast and transform $\mathcal{I}'_{RGB}$
Get CH_{score} of $\mathcal{I}_{YCbCr}$
Local cluster set $\mathcal{L} \leftarrow$ K-means($\mathcal{M} \cap \mathcal{I}_{YCbCr}, k = 2$)
Global cluster set $\mathcal{G} \leftarrow$ K-means($\mathcal{I}_{YCbCr}, k = f \cdot CH_{score}$)
Split clusters in $\mathcal{G}$ using $\mathcal{E}$
Vessel cluster v, Cortex cluster $c \leftarrow$ Split $\mathcal{L}$ by brightness
for $i = 1, N_{iters}$ **do**
  Init set of extended vessel clusters $\mathcal{N}_v \leftarrow \emptyset$
  Init set of extended cortex clusters $\mathcal{N}_c \leftarrow \emptyset$
  Get the set of clusters $\mathcal{N} \subset \mathcal{G}$ neighboring $\mathcal{M}$
  foreach $c_n \in \mathcal{N}$ **do**
    if $D_C(c_n, v) < \sigma(v)$ **then** // Equations (S1) and (S2)
      $\mathcal{N}_v := \mathcal{N}_v \cup \{c_n\}$
  Get the set of clusters $\mathcal{N} \subset \mathcal{G}$ neighboring $\mathcal{N}_v$
  foreach $c_n \in \mathcal{N}$ **do**
    if $D_C(c_n, c) < \sigma(c)$ **then** // Equations (S1) and (S2)
      $\mathcal{N}_c := \mathcal{N}_c \cup \{c_n\}$
  $\mathcal{M} := \mathcal{M} \cup \{(\mathcal{N}_v \cup \mathcal{N}_c) \cdot 1/i\}$
$\mathcal{M}_{ref} \leftarrow$ Apply mode filter to $\mathcal{M}$

Describing the method in more detail, the first step is to crop the RGB images to obtain a region of interest (ROI) centered on the manual annotations, leaving the same margin of

108 pixels mentioned in Section 3.1.7 in the original manuscript, but in this case to ensure that the entire cerebral surface is included in the ROI. The main contours of the image are then detected using the Canny algorithm [1]. These contours are intended to capture relevant edges present in the image, such as those separating the dura mater from the cortex. Since some of the clusters produced by K-means may contain pixels from both the dura mater and brain, the acquired edges are used to split any cluster they pass through. In this way, the annotated mask is less likely to spread excessively through the dura samples.

As indicated above, the underlying principle of the performed method is to compare the pixels belonging to the manually annotated area with their surrounding regions to determine whether they are of the same type. So after converting the RGB image into the YCbCr colorspace and adjusting its contrast, two types of clustering are performed:

- Local clustering, in which the labeled region is divided into two clusters with the intention of separating the pixels of the cerebral cortex from the pixels of the blood vessels.
- Global clustering, by segmenting the entire image using a number of clusters N estimated using the Calinski-Harabasz (CH) score [2]. For each image, the interval between 5 and 20 clusters is evaluated, selecting the number of clusters that yields the highest CH score. In order to cautiously expand the manually annotated regions by these clusters, the number N suggested by the CH score is multiplied by a given factor f, thus performing an intentional over-segmentation. In this work, the factor f is empirically found to produce excessively atomised clusters above a value of 3, which makes mask expansion problematic. It is therefore set to 3.

The comparison between clusters belonging to the annotated area and unknown surrounding clusters is performed using the cosine dissimilarity D_C, taken as $1 - S_C$ where S_C is the cosine similarity. Given an unlabeled cluster B and the samples that conform to it as $B = \{b_0, b_1, ..., b_N\}$ with $b_i \in \mathbb{R}^3$, and the centroid $\bar{A} \in \mathbb{R}^3$ of a labeled cluster A, the average cosine dissimilarity $D_C(\bar{A}, B)$ is calculated as in Equation (S1):

$$D_C(A, B) = \frac{1}{N} \sum_{i=1}^{N} \left(1 - \frac{b_i \cdot \bar{A}^T}{\|b_i\| \|\bar{A}\|}\right) \tag{S1}$$

The value obtained is then compared with the standard deviation, also based on cosine dissimilarity, of the cluster A with respect to its set of samples $\{a_0, a_1, \ldots, a_M\}$ and calculated as in Equation (S2):

$$\sigma(A) = \frac{1}{M} \sum_{i=1}^{M} \left(1 - \frac{a_i \cdot \bar{A}^T}{\|a_i\| \|\bar{A}\|}\right) \tag{S2}$$

The unlabeled clusters that surround the annotated mask are evaluated in such a way that if $D_C(A, B) < \sigma(A)$ the cluster B is considered to be of the same kind of cluster A and, therefore, included in the refined mask.

As explained above, the masked first expands by comparing neighboring clusters with the one taken as vascular tissue. Once all its neighbors have been evaluated, the neighboring clusters of the updated mask are compared to the labeled cluster considered as cerebral cortex. Given the deliberately slow pace of the annotation expansion to avoid spreading across the craniotomy site, this process must be iterative. In particular, 4 iterations provide satisfactory results for the majority of images. In addition, the probability assigned to the updated areas of the refined mask is decreased proportionally to the number of iterations. Thus, the regions included in the first iteration have a probability of 1, while the regions added in the fourth iteration have a probability of 1/4, reducing the confidence in the added regions as they are further away from the original annotations.The reason for this

approach is to give the refined labels a smoothing effect, which may help improve the generalizability of the model [3]. The mask contour obtained is finally softened with a mode filter with a 15-pixel size squared kernel.

S2. Cortical vessel pseudo-label generation

In this section, further details for the parameterization of the vascular pseudo-label extraction process are provided. Following the method introduced in Section 3.2.3 of the manuscript, the two linear operators are applied using a sliding window approach, outputting the intensity captured by each of them into two different images. Before being combined, both images are thresholded using a different value for each image. In order to apply the complete methodology, this process requires the parameterization of the band to be selected from the HS cube, the two kernel sizes, and the two thresholds to be applied. The setting of these parameters should lead to a maximum percentage of ground truth blood vessel samples and a minimum amount of healthy and tumor samples included in the detected contours. Under these conditions, the Optuna optimization framework [4] is employed for the optimization process using the same set of images reserved for training and validation in Section 4 of the manuscript. The kernel sizes are tested in the range of $[5, 13]$ for the smaller one and $[19, 25]$ for the bigger one whereas the thresholds applied to the result of each linear operator are explored in the $[5, 100]$ and $[700, 1000]$ range respectively. After 150 trials, the set of all the combinations of parameters explored is arranged according to the percentage of detected vessel pixels and the percentage of erroneously included brain surface samples. Only combinations of parameters that obtained an average detection rate of vascular samples greater than 97% in the defined set of images are considered. Among them, the set of parameters with the lowest percentage of segmented healthy and tumor samples is selected. The final optimized values of each parameter are shown in Table S1. As a complementary validation of the optimization process, it is worth mentioning that the selected spectral band corresponds to 762.7 nm, which, according to [5], matches the peak of the molar extinction coefficient in the near-infrared spectrum of deoxygenated hemoglobin. This aspect can be justified by the abundance of veins among the vessels to be detected.

Table S1. Optimized parameters using the Optuna framework to generate cortical vessel pseudo-labels with the HS dataset. Both kernel parameters indicate the size in pixels per size of the linear operators contained in the kernel.

Band	Kernel 1	Kernel 2	Thresh. 1	Thresh. 2
7	7	25	40	900

The generation of blood vessel pseudo-labels for the RGB dataset is performed through the same method described above for the HS images. Since RGB images do not have any annotations for vascular tissue, the same set of parameters of Table S1 is applied with the exception of *Thresh. 1*. It was observed that the higher contrast of the RGB images made them more likely to produce artefacts in the output pseudo-label image. Therefore, it was necessary to manually increase *Thresh. 1* value to 80 to make it less permissive. In order to proceed without modifying the rest of the parameters, it is fundamental that the RGB images are cropped and rescaled so that they have the same resolution as the HS images. Taking the manually annotated regions described in Section 3.1.7 of the manuscript, for each image, an area with the same aspect ratio as the HS images is cropped, leaving a minimum margin of 5% of the annotation height between the end of the annotations and the crop boundaries. The resulting image is then resized to 217×409 pixels. After the

resolution of the RGB images is adjusted, they are converted to the YCbCr colorspace to select the luma channel as the grayscale input image.

The last step in the generation of RGB vessel pseudo-labels consists of using the refined annotations obtained in Section 3.2.1 of the manuscript, to select only the contours that lie within the refined mask and also to remove dura mater borders that could have been detected as blood vessels.

S3. HSI ground truth densification and background complementation

The background areas extracted in Section 3.2.4 of the original manuscript rely on a series of steps whose implementation details are provided in this section. In order to reliably compose the background areas, the same clustering based strategy used in the RGB brain cortex label refining (Section 3.2.1 of the manuscript) is applied. The number of clusters k for the K-means algorithm, is calculated using the CH score for the complete HS image, exploring the range between 5 and 50 clusters. The number of clusters yielding the maximum score is then multiplied by a factor $f = 3$ to induce an intentional oversegmentation, resulting in a final value of $k = 35$, which is the average of the individual values obtained using the training and validation set of images. On the other hand, labeled samples are grouped in 4 clusters, one for each class. The cosine similarity between the centroids of the clusters belonging to the labeled and unlabeled regions is then calculated. The unlabeled clusters are ranked according to their dissimilarity to labeled clusters, so that if two or more of the four clusters belonging to the labeled samples have the same unlabeled cluster as the most dissimilar, that unlabeled cluster is considered to be part of the region outside the cerebral cortex. From this ranked list, selecting the top eight most dissimilar unlabeled clusters empirically ensures that no brain surface pixel is included in the background mask.

References

1. Canny, J. A Computational Approach to Edge Detection. *IEEE Transactions on Pattern Analysis and Machine Intelligence* **1986**, *PAMI-8*, 679–698. https://doi.org/10.1109/TPAMI.1986.4767851.
2. Caliński, T.; Harabasz, J. A dendrite method for cluster analysis. *Communications in Statistics* **1974**, *3*, 1–27, [https://www.tandfonline.com/doi/pdf/10.1080/03610927408827101]. https://doi.org/10.1080/03610927408827101.
3. Szegedy, C.; Vanhoucke, V.; Ioffe, S.; Shlens, J.; Wojna, Z. Rethinking the Inception Architecture for Computer Vision. *CoRR* **2015**, *abs/1512.00567*, [1512.00567].
4. Akiba, T.; Sano, S.; Yanase, T.; Ohta, T.; Koyama, M. Optuna: A Next-generation Hyperparameter Optimization Framework, 2019. https://doi.org/10.48550/ARXIV.1907.10902.
5. Scheeren, T.; Schober, P.; Schwarte, L. Monitoring tissue oxygenation by near infrared spectroscopy (NIRS): background and current applications. *Journal of clinical monitoring and computing* **2012**, *26*, 279–287.
